# Chimeric Antigen Receptor (CAR)-Specific Monoclonal Antibody to Detect CD19-Specific T Cells in Clinical Trials

**DOI:** 10.1371/journal.pone.0057838

**Published:** 2013-03-01

**Authors:** Bipulendu Jena, Sourindra Maiti, Helen Huls, Harjeet Singh, Dean A. Lee, Richard E. Champlin, Laurence J. N. Cooper

**Affiliations:** 1 Division of Pediatrics, The University of Texas MD Anderson Cancer Center, Houston, Texas, United States of America; 2 The University of Texas Graduate School of Biomedical Sciences, Houston, Texas, United States of America; 3 Department of Stem Cell Transplantation and Cellular Therapy, The University of Texas MD Anderson Cancer Center, Houston, Texas, United States of America; National Cancer Institute, NIH, United States of America

## Abstract

Clinical trials targeting CD19 on B-cell malignancies are underway with encouraging anti-tumor responses. Most infuse T cells genetically modified to express a chimeric antigen receptor (CAR) with specificity derived from the scFv region of a CD19-specific mouse monoclonal antibody (mAb, clone FMC63). We describe a novel anti-idiotype monoclonal antibody (mAb) to detect CD19-specific CAR^+^ T cells before and after their adoptive transfer. This mouse mAb was generated by immunizing with a cellular vaccine expressing the antigen-recognition domain of FMC63. The specificity of the mAb (clone no. 136.20.1) was confined to the scFv region of the CAR as validated by inhibiting CAR-dependent lysis of CD19^+^ tumor targets. This clone can be used to detect CD19-specific CAR^+^ T cells in peripheral blood mononuclear cells at a sensitivity of 1∶1,000. In clinical settings the mAb is used to inform on the immunophenotype and persistence of administered CD19-specific T cells. Thus, our CD19-specific CAR mAb (clone no. 136.20.1) will be useful to investigators implementing CD19-specific CAR^+^ T cells to treat B-lineage malignancies. The methodology described to develop a CAR-specific anti-idiotypic mAb could be extended to other gene therapy trials targeting different tumor associated antigens in the context of CAR-based adoptive T-cell therapy.

## Introduction

**Figure 1:**
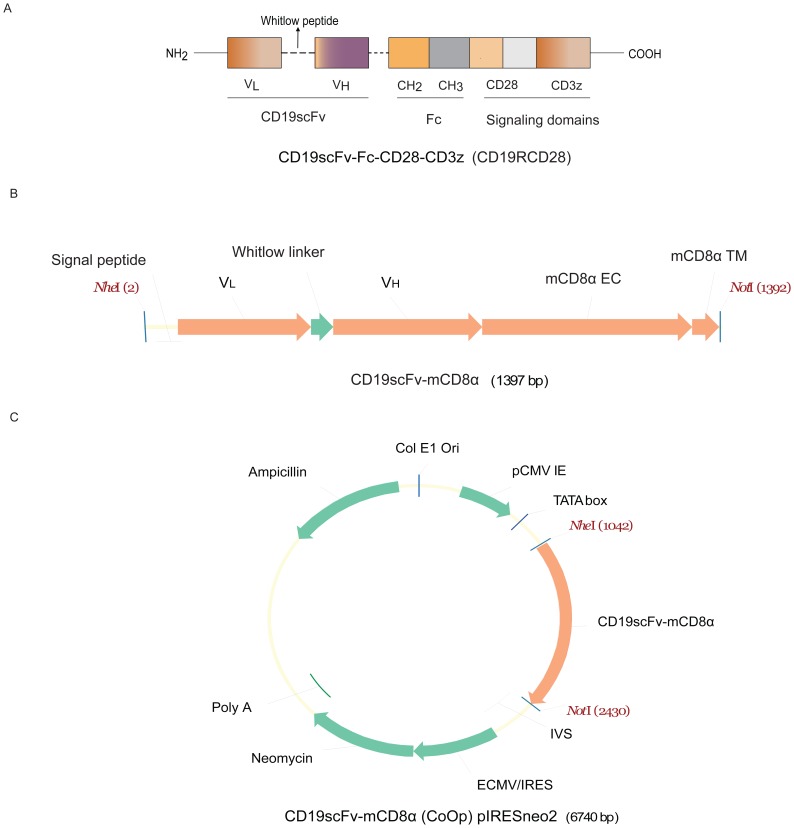
Schematic of DNA plasmids used for electroporation and expression of transgenes. (A) Constructs showing components of CD19-specific CAR (CD19RCD28). The scFv is derived from mAb clone FMC63 that binds human CD19 and was generated by fusing the V_L_ and V_H_ regions via a “Whitlow” linker peptide. The scFv was attached to modified human IgG_4_ hinge and CH_2_-CH_3_ regions that was fused to the CD28 (transmembrane and cytoplasmic) and CD3ζ (cytoplasmic) domains [24] (B) DNA plasmid design CD19scFv-mCD8α used to express the transgene on L cells for immunization and screening. The CD19-specific scFv was generated as described above, mCD8α EC and TM represents mouse CD8α extracellular and transmembrane domains respectively. (C) Map of destination vector pIRESneo2 (Clontech) where the cDNA for the fusion protein CD19scFv-mCD8α is cloned into *Nhe*I and *Not*I restriction enzyme sites. Expression cassette shows ColE1, colicin E1 (origin of replication); pCMV IE, human cytomegalovirus promoter/enhancer; ECMV IRES, encephalomyocarditis virus internal ribosome entry site which permits translation of two open reading frames from one messenger RNA, and poly A (polyadenylation) signal of the bovine growth hormone. Plasmid confers resistance through ampicillin and after electroporation the cells expressing the transgene of interest are selected on cytocidal concentration of drug neomycin sulfate (G418).

Genetically modified T cells engineered to express a tumor-specific chimeric antigen receptor (CAR) have been infused initially with modest [1], [2], [3] and recently significant anti-tumor effects [4], [5], [6], [7], [8]. The prototypical CAR uses an extracellular domain to directly dock to a cell surface molecule which is usually a tumor-associated antigen (TAA). The specificity of a CAR is typically derived from a scFv region assembled from the antigen-binding region of a TAA-specific monoclonal antibody (mAb). The components of the 2^nd^ generation CD19-specific CAR currently used in our clinical trials (Clinical trial.gov Id# NCT00968760, NCT01497184, NCT01362452), designated CD19RCD28 are shown in Fig. 1A. The scFv from mouse mAb clone FMC63 [9] is fused in frame to an extracellular scaffold (*e.g*., human immunoglobulin hinge and Fc regions or hinge and constant regions from human CD8α) to promote oligomerization after binding TAA which contributes to CAR-dependent activation via one or more signaling motifs in the endodomain. Multiple Phase I clinical trials designed to treat B-cell malignancies redirect the specificity of CAR^+^ T cells based on the CD19-specific mAb clone FMC63 [9], [10].

Investigators measure the persistence of adoptively transferred T cells in vivo to assess the therapeutic potential of CAR^+^ T cells [5], [11]. The most common approach to assessing survival of infused CAR^+^ T cells is quantitative PCR using CAR-specific primers [4], [12]. However, this technique does not allow retrieval of genetically modified T cells from the recipient for multi-parameter analyses. What is needed and is provided here is a mAb to specifically detect and isolate CD19-specific CAR^+^ genetically modified T cells. In addition to informing on persistence, a CAR-specific mAb can be used for in-process testing during manufacture to assess expression of the transgene, such as after electro-transfer of *Sleeping Beauty* (SB) or *piggyBac* DNA plasmids coding for CD19RCD28 [13], [14], [15], [16].

We report here an approach for developing an anti-idiotype (anti-Id) mAb with specificity for CD19-specific CARs that could be extended to other CARs targeting alternative TAAs. The mouse mAb (clone no. 136.20.1) characterized in this paper recognizes CD19-specific CARs derived from anti-human CD19 mAb (clone FMC63). We show specificity of this mAb for CD19-specific CAR using (i) flow cytometry, (ii) western blotting, (iii) transmission electron microscopy (TEM), and (iv) immunocytochemistry. The binding of this anti-Id to the scFv region of CD19RCD28 was demonstrated using a panel of CAR^+^ T cells and inhibition of CD19-dependent cytolysis as observed by chromium release assay (CRA) and video time lapse microscopy (VTLM).

## Materials and Methods

### Ethics Statement

All experimental procedures pertaining to the use of animals were performed according to the U.T. MD Anderson Cancer Center Institutional Animal Care and Use Committee (IACUC) guidelines and were carried out by the MD Anderson Hybridoma core lab (IACUC approval no. 11-071-2533). All efforts were made to reduce suffering to the animals caused by any experimental procedure. Isoflurane (2–5%) were used as anesthesia whenever required. Details of animal care, anesthesia use and sample collection are provided in separate paragraph. For the peripheral blood mononuclear cells (PBMC) used in the study, blood samples were obtained from healthy volunteers under the protocol title “Acquisition of peripheral blood from healthy volunteers” that aimed to investigate the immunobiology of lymphocytes such as T cells in general and genetically modified T cells in particular (approval obtained from U.T. MD Anderson Cancer Center Institutional Review Board protocol no. LAB07-0296). Healthy volunteers donated blood only after written consent was obtained and the study objectives were explained.

### Cells

Cell lines were obtained from American Type Culture Collection (ATCC) center unless otherwise stated. L cells, (a mouse adherent fibroblast cell line derived from C3H/An strain; ATCC no. CRL-2648), Jurkat cells (ATCC no. CRL-1990), NSO cells (Sigma Aldrich no.****85110503), NALM-6 pre-B ALL cell (DSMZ no. ACC 128), EL4 murine lymphoma T cell line (ATCC no. TIB-39), CD19^+^ EL4 (genetically modified to express truncated human CD19) [17], Daudi co-expressing EGFP and β_2_-microglobulin (Daudi β_2_m), were all cultured in complete media (CM) defined as RPMI 1640 (Hyclone) supplemented with heat inactivated 10% fetal bovine serum (FBS) (Hyclone) and 2 mM L-Glutamine (Gibco-Invitrogen). Genetically modified cells were selected in various cytocidal concentrations of neomycin sulfate G418 (Invivogen) at 0.8 mg/mL for NSO cells, 0.9 mg/mL for L cells and 1 mg/mL for Jurkat cells [13]. K562 were transduced with lentivirus to co-express CD64, CD86, CD137L and a membrane-bound IL-15 (mIL15) to generate artificial antigen presenting cells (aAPC clone no. 4) [8], [13], [14], [16]. Primary human T cells from PBMC were genetically modified to express CD19RCD28 CAR using the *Sleeping Beauty* (SB) transposon/transposase system and propagated ex vivo in a CAR-dependent manner on CD19^+^ aAPC (K562, clone no. 4) in CM supplemented with soluble recombinant IL-2 (Novartis/Chiron) at 50 IU/mL and IL-21 at 30 ng/mL (Peprotech) [16], [18].

Cell Surface xpression of CD19-specific scFvV_L_ (amino terminus) and V_H_ (carboxyl terminus) regions were derived from anti-human CD19-specific mAb clone FMC63 [9]. The scFv was formed by joining the V_L_ and V_H_ with 18 amino acid (AA) Whitlow peptide linker (GSTSGSGKPGSGEGSTKG) [19]. This binding domain denoted as CD19scFv was then fused in frame with mouse CD8α extra-cellular domains (AA 28–196) and transmembrane domains (AA 197–217) (Swiss-Prot No.P01731 www.expasy.org) to create the fusion protein CD19scFvmCD8α. The cDNA representing the fusion protein was mouse codon optimized and synthesized by a commercial vendor (Fig. 1B, GENEART). The nucleotide sequence of CD19scFvmCD8α was verified and the cDNA was cloned into plasmid pIRESneo2 (Clontech cat no. 6938-1) to generate DNA vector CD19scFvmCD8αpIRESneo2 (Fig. 1C). Purified vector CD19scFvmCD8αpIRESneo2 DNA (endotoxin level <4 EU/mL) at concentrations of 1 µg per 10^6^ of L cells and 5 µg per 10^6^ Jurkat and NSO cells, were electro-transferred by a Nucleofector device (model no. AAD-1001, Lonza) employing Nucleofector kit R and program X-005 for L cells and human T cell Nucleofector kit and program U-14 for Jurkat and NSO cells. Electroporated cells were plated in 6-well culture plates (Corning) containing complete media (CM), rested for 4 hours, and then added with drug neomycin sulfate (G418) as mentioned. Cells were cultured in an incubator (5% CO_2_ and 37°C) and at 24 to 48 hours the transient expression of CD19scFvmCD8α was assessed by flow cytometry using PE-conjugated anti-mouse CD8α antibody (BD Biosciences). Drug-resistant clones of L cells were propagated to confluence and harvested by trypsinization using 0.05% Trypsin-EDTA (Gibco-Invitrogen). Cells with highest expression level of CD19scFvmCD8α were sorted on a FACSAria sorter (BD Biosciences) using anti-mouse CD8α-PE (BD Biosciences) as detection antibody. Sorted cells were numerically expanded further in the presence of neomycin sulfate (G418). Transgene expression was confirmed by flow cytometry. One L cell (clone no. 12) with stable highest expression of transgene was chosen and expanded further for immunization purposes. Similarly, Jurkat and NSO cells expressing CD19scFv were generated to screen hybridoma clones.

### Animal Care, Immunization, Lymphocyte Collection

All the experiments pertaining to the animal use were performed as per the recommendations of the U.T. MD Anderson Cancer Center Institutional Animal Care and Use Committee (IACUC approval no. 11-071-2533). Two BALB/c mice (NCI, Frederick) were immunized in the footpads, each with 5×10^6^ L cells expressing CD19scFv-mCD8α (clone no. 12) suspended in 50 µL PBS. No adjuvant was used for immunization. The mice were restrained manually (for less than 2 minutes) for cell injection or anesthetized by isoflurane (2–5%) whenever required. Each mouse received a total of 6 injections at every 3 days interval. At the end of 5^th^ immunization, mice were anesthetized by isoflurane (2–5%) inhalation and then blood was drawn from the tail vein. Sera were used to evaluate the antibody titer in an indirect ELISA as described (Supplementary info method S1). On Day 18 a mouse with highest titer was chosen for lymphocyte collection and fusion. Mice were euthanized by CO_2_ inhalation and then popliteal lymph nodes were dissected for collection of lymphocytes.

### Generation of Hybridomas

A schematic outlining the steps involved for development and isolation of a mAb with specificity for the CD19scFv region in CD19RCD28 CAR is shown in supplementary info Fig. S1. In brief, lymphocytes were washed with RPMI 1640 and then fused with a myeloma partner SP2/0 (ATCC no. CRL1581) at a lymphocyte to myeloma ratio of 1∶0.8. Hybridomas were obtained by standard polyethylene glycol (PEG-1450, Sigma-Aldrich) mediated fusion technology. Fused cells were grown in 96-well tissue-culture plates for 10 days in HAT selection media (Sigma-Aldrich) supplemented with 10% hybridoma cloning supplement (HCS) (GE/PAA Lab Inc). Hybridoma clones were screened on day 10 to evaluate for the presence of antibody.

### Screening Hybridoma Clones

A solid phase ELISA was used to screen for hybridoma clones producing mAb specific for CD19scFv. Details of screening hybridoma clones are provided in Supplementary method S1. Monoclonal antibodies were purified by standard protein G or protein A columns. Details of purification and conjugation procedures are shown in Supplementary method S2. One hybridoma clone (no. 136.20.1) was selected for characterization.

### Flow Cytometry

For detecting surface expression of CAR, about 0.25×10^6^ cells were suspended in 0.1 mL of FACS buffer (2% FBS in 1X PBS) and stained with antibodies conjugated to fluorochromes (Table S1). Following blocking for 30 minutes at 4°C with 2% normal mouse sera (Jackson ImmunoResearch) in FACS buffer and washing twice with FACS buffer, the samples were incubated on ice with primary antibodies or conjugated antibodies for 30 minutes. Data were acquired on a FACSCalibur (BD Biosciences) using CellQuest software, version 3.3 (BD Biosciences). Dot plot analyses or MFI (median fluorescence intensity) was calculated by either FCS Express version 3.00.007 (Thornhill) or FlowJo software (version 7.6). To determine the sensitivity of anti-CD19scFv mAb (clone no. 136.20.1), CD19-specific CAR^+^ T cells were mixed with PBMC obtained from healthy donors (CAR^neg^ cells) at varying cell ratios (1∶10–1∶10,000 CAR^+^ T cell to PBMC). AlexaFluor-647 conjugated mAb (clone no. 136.20.1) was used to detect CAR^+^ T cells by flow cytometry.

### Binding Assay

Binding activity of purified anti-CD19scFv mAb (clone no. 136.20.1) was determined by an indirect ELISA. In brief, wells of a 96-well plate (Thermo Scientific Nunc) were coated with 100 ng of following mAbs: anti-human CD19 mAb (clone FMC63, Millipore), anti-human CD19 mAb (clone MHCD1900, Gibco-Invitrogen), anti-human CD20 mAb (clone no. 1F5.1D4.1 derived by us from clone 1F5, clone no. HB-9645, ATCC), human IgG (Jackson ImmunoResearch) in 0.1 mL of coating buffer (100 mM NaHCO_3_ pH 8.6). The plates were incubated at 37°C for 1 hour and then washed three times with PBST (1XPBS with 0.05% tween-20). Blocking was achieved with 300 µL of 5% BSA in PBST. A dose curve was generated using different concentrations of purified anti-CD19scFv primary mAbs (2–30 µg/mL) and detected by secondary antibody goat anti-mouse IgG Fc HRP (Sigma-Aldrich). Absorbance was read at 450 nm using a microplate reader (Victor 2030, Perkin Elmer).

### Western Blot

Reactivity of anti-CD19ScFv mAb (clone no. 136.20.1) to SDS-denatured protein was tested by western blot analysis. 10^7^ CAR modified or unmodified control T cells were lysed using RIPA buffer (50 mM Tris, pH 7.5, 150 mM NaCl, 0.1% SDS, 0.5% Sodium Deoxycholate, 1% TritonX100, 1 mM PMSF) containing protease inhibitor tablet as per manufacturer’s instructions (Roche Applied Science). Protein concentration was determined by a BCA kit (Thermo Scientific Pierce). 10 µg of total protein obtained from each cell lysates were electrophoresed under denaturing condition on a 4–20% gradient gel (Biorad) followed by electrobloting onto a PVDF membrane using criterion blotter (Biorad). PVDF membrane was blocked using 5% skim milk in PBST, and then incubated with primary anti-CD19ScFv mAb (clone no. 136.20.1). Binding was detected by goat anti-mouse IgG Fc HRP (Sigma-Aldrich) enhanced with ECL Westfemto™ substrate (Thermo Scientific Pierce). Images of blots were acquired on a gel doc image system using versa doc Quantityone™ software (Biorad).

### Microscopy

For confocal microscopy genetically modified T cells expressing CD19RCD28 CAR were fixed in 4% paraformaldehyde (EMS Inc.) and stained with AlexaFluor 647 conjugated anti-CD19scFv mAb (clone 136.20.1) at 1∶500 using standard protocol. Labeled cells were added with ProLong gold anti-fade reagent with DAPI (Gibco-Invitrogen) and viewed under a confocal microscope (Leica Microsystems). Cells were also fixed in neutral buffer formalin for H&E staining and visualization of CAR+ T cells by DAB staining (Details are provided as supplementary method S3). For TEM, CAR^+^ T cells were fixed in 1% glutaraldehyde (Sigma-Aldrich) followed by washing with PBS. The cells were then incubated in 20 mM glycine-PBS followed by washing with PBS-BSA buffer (20 mM phosphate, 150 mM NaCl pH 7.4 containing 0.5% BSA and 0.1% gelatin). NANOGOLD antibody conjugates (Supplementary method S2) were diluted 1∶50 in PBS-BSA buffer and incubated with CD19-specific CAR^+^ T cells for 15 min followed by washing with the same buffer to remove unbound conjugates. Finally, NANOGOLD bound cells were fixed with 1% glutaraldehyde and stored in 4°C until further use. Before sectioning cells were subjected to an alcohol dehydration steps (5 to 100% graded alcohol separated in 5 steps) followed by embedding in epoxy resin. The resin embedded cells were then cut into sections (1 µm to 100 nm thickness) in an ultra-microtome (Leica Microsystem). Sections were drawn onto a formavar coated copper grid. Staining was done by 2% uranyl acetate and then air dried. Unstained samples underwent silver enhancement method (Nanoprobes) so that surface bound NANOGOLD particles could be visualized. High magnification images were acquired by a 1 k×1 k CCD camera attached to a JEM-1200 EX60KV electron microscope (JEOL).

### Chromium Release Assay (CRA)

The functionality of anti-CD19scFv mAb was assessed in a CD19-specific CAR^+^ T cell mediated cytolysis in a standard 4 hour chromium (^51^Cr) release assay (CRA) employing parental CD19^neg^ EL4, CD19^+^ EL4, CD19^+^ Daudiβ_2_m and CD19^+^ NALM-6 as targets [13], [16]. Effector cells (ex vivo expanded CD19RCD28 CAR^+^ T cells harvested on day 28) were incubated with various concentrations of purified mAb (clone 136.20.1) at 10-fold serial dilution (maximum dose at 250 µg/mL and minimum at 1.25 µg/mL) for 20 min at 4°C, followed by washing with RPMI 1640 to remove unbound antibodies. The antibody-bound effector cells were incubated with ^51^Cr labeled target cells in a clear v-bottom 96-well plate (Corning) at 37°C. Release of ^51^Cr was quantified in a micro-plate scintillation counter TopCount NXT (Perkin-Elmer). Specific lysis was calculated for effector to target cell (E:T) ratios in the presence and absence of anti-CD19scFv mAb. Data are reported as mean ± standard deviation (SD).

### Video Time Lapse Microscopy (VTLM)

CD19-specific T cells expressing CD19RCD28 CAR were washed in sterile 1× PBS and stained for 30 min at room temperature using AlexaFluor 647-conjugated to anti-CD19ScFv mAb (clone no. 136.20.1) at 2 µg mAb per 100,000 cells and then washed three times with 1× PBS. The CAR^+^ T cells were mixed together with EGFP^+^ Daudiβ_2_m at an effector to target ratio of 2∶1. A cell pellet was made by brief centrifugation and then re-suspended in media RPMI 1640 and put in one of the chamber of a 35 mm culture dish (Nikon). This was kept in the specimen chamber of Biostation IM (Nikon) and allowed to settle for 10 min. CCD camera focal planes were fixed and the effector to target cell dynamics were assessed for 2 to 12 hours using imaging software (IM-Q, v2.1.2.136, Nikon). As control, APC-conjugated goat anti-human IgG Fcγ chain specific F(ab′)_2_ fragment antibody (Jackson ImmunoResearch) was used to bind CAR^+^ T cells and the co-culture was set up as described.

## Results

### Generating Anti-ScFv mAb Recognizing CD19RCD28 CAR

We immunized BALB/c mice using L cells (derived from C3H/An mouse strain) genetically modified to express the scFv region in a CD19-specific CAR (Fig. S1). The scFv was derived from FMC63 (encoded in DNA plasmid CD19scFv-mCD8α) and stably expressed (>90% homogenous expression after single-cell sorting) on the surface of neomycin-resistant genetically modified L cells which was used to immunize allogeneic mice. The murine CD8α trans-membrane and extra-cellular region was used to display scFv on surface and to detect expression (Fig. 1B and 1C). Genetically modified L cells were injected in mice at 3 day interval and high titer sera (OD values ∼5 times above background at 1∶2,700 dilution) was obtained after five successive immunizations. Hybridomas were generated and ELISA was used to initially select 23 “high binders” (OD_450_>1.5) and 15 “moderate binders” (OD_450_∼1.00–1.5) to advance to the second round of screening. Flow cytometry using Jurkat and NSO cells genetically modified with CD19scFv-mCD8α was used to cull the number of candidate hybridomas to 12. One clone was selected (no. 136.20.1) that could efficiently bind CD19RCD28 expressed cells by flow cytometry. The absence of adjuvant at the time of immunization did not apparently affect the antibody affinity maturation process as we obtained mAb clones predominantly of IgG_2a_ sub-class. Immunization by genetically modified L cells provides an efficient method for eliciting desirable anti-idiotype immune response.

### Specificity of mAb (Clone No. 136.20.1) for CD19scFv

**Figure 2:**
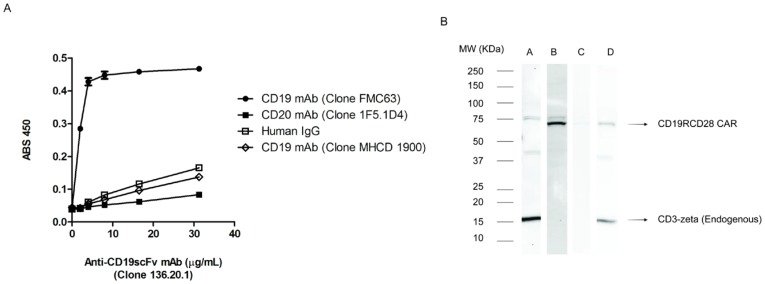
Specificity of anti-CD19scFv mAb. (A) Solid phase ELISA shows specificity of mAb (clone no. 136.20.1) as it binds to parental monoclonal antibody (FMC63) with background binding to other antibodies (*e.g.*, CD20-specific mAb or a different CD19-specific mAb). Purified human IgG served as a negative control. (B) Western blot shows clone 136.20.1 detects of CAR protein in T cells genetically modified to express CD19RCD28. Lane A: Unmodified control T cells show endogenous CD3ζ (14 kDa) as detected by commercial CD3ζ-specific antibody and absence of CAR. Lane B: CAR^+^ T cells show CAR-specific band at 75 kDa as detected by mAb (clone 136.20.1). Lane C: CAR^+^ T cells show absence of CAR-specific band when the blot is treated with primary antibody after blocking (clone 136.20.1 was blocked with molar excess (1∶5) of parental antibody FMC63). Lane D: CAR^+^ T cells show the presence of CAR (∼75 kDa) and endogenous CD3ζ (14 KDa) as detected by commercial CD3ζ-specific antibody.

**Figure 3:**
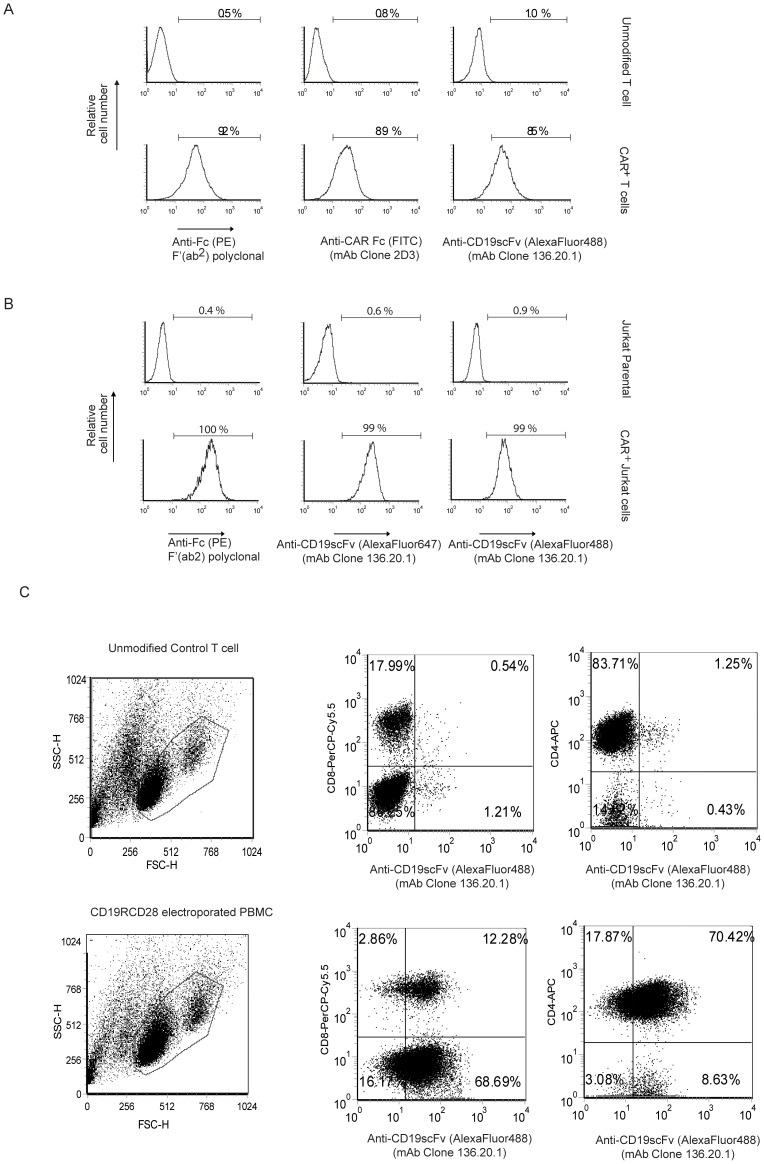
Detection of CD19-specific CAR on the surface of genetically modified cells. (A) CAR^+^ T cells were electroporated with *Sleeping Beauty* system and propagated on aAPC. Upper row: Unmodified T cells as back ground control; Bottom row: CD19RCD28^+^ T cells (labeled as CAR^+^ T cells) detected by flow cytometry using clone no. 136.20.1, a commercially-available antibody (clone H10104, Invitrogen) that binds to the CAR hinge/Fc scaffold, and our Fc scaffold-specific mAb (clone 2D3). (B) Jurkat cells were genetically modified to express CD19RCD28 was detected by clone no. 136.20.1 conjugated to Alexa-Fluor 488 and Alexa-Fluor 647 similar to commercial antibody (clone H10104). (C) Multi-parameter flow cytometry analysis of T cells genetically modified to express CD19RCD28. Sequential staining was performed using anti-CD19scFv mAb and/or anti-CD4, anti-CD8 mAb in combination.

The specificity of mAb (clone no. 136.20.1) towards CD19scFv was initially evaluated by an indirect ELISA with wells coated with purified mAbs. Except for parental anti-human CD19 mAb (FMC63) all other antibodies exhibited negligible cross reactivity to CD19scFv specific mAb (Fig. 2A). Anti-CD19scFv binds to its parental mAb at concentration as low as 2 µg/mL with binding saturated at 4 µg/mL. The specificity of this mAb towards clone FMC63 was confirmed as it did not cross react to another anti-human CD19 antibody (Invitrogen, clone MHCD 1900). Further, it did not bind to purified human IgG and CD20-specific mAb. The ability of mAb (clone no. 136.20.1) to detect CD19^+^ CAR protein was verified by western blot using whole protein lysates obtained from ex vivo-propagated CD19-specific CAR^+^ T cells. MAb (clone no. 136.20.1) detected a ∼75 kDa protein in CD19RCD28 modified cell lysates run in reducing SDS-PAGE and electroblotted onto PVDF membrane (Fig. 2B, lane B). The specificity was verified on parallel by a commercial available CD3ζ antibody which detects CD3ζ signaling domain in CD19RCD28 expressing CAR (Fig. 2B, lane D) and endogenous CD3ζ in unmodified control T cells (Fig. 2B, lane A). The ability of this mAb to detect the surface expression of CD19^+^ CAR was verified by flow cytometry analysis using CD19RCD28-expressing primary T cells and Jurkat cells (Fig. 3A) and (Fig. 3B) respectively. MAb (clone 136.20.1) detected expression of CD19scFv on surface of genetically modified T cells (85%) as well as in Jurkat cells (99%) which matches with detection level of anti-Fc antibodies that binds to CH_2_–CH_3_ stalk of the CAR. Indeed, the staining pattern was compared with commercial IgG-γ chain specific antibodies (Fc-PE Invitrogen) that binds to CH_2_–CH_3_ region of the CAR and by mAb (clone no. 2D3 developed in-house) which binds to the CH_2_–CH_3_ hinge region of the CAR (Fc-FITC) [13]. A panel of CAR^+^ T cells that target different TAAs (CD33, CD123, ROR1, HERV-K) were used to verify if the clone no. 136.20.1 cross reacts with any of these CAR transgenes. Each CAR construct was expressed from *Sleeping Beauty* vector backbone and contains different signaling endodomains in addition to the common IgG_4_-derived hinge/CH_2_–CH_3_ linker. It did not cross react with any of other CARs tested other than those with specificity for CD19 (Fig. S2). No background binding to mock-electroporated control T cells was observed. Additional evidence supporting specificity of mAb (clone no. 136.20.1) are provided as supplementary data (Fig. S3 and Fig. S4 **,** kindly provided by Dr. Gianpietro Dotti at Baylor College of Medicine, Houston and Dr. Stephen Forman at City of Hope National Medical Center). In both cases CAR scaffold differs either by presence of human CD4 transmembrane region or IgG_1_ Fc stalk that connects the scFv with the signaling endodomains. Furthermore, our mAb has been used to detect CD19-specific CAR^+^ T cells that employ an extracellular domain derived from CD8α [5], [20]. These results demonstrate that irrespective of extra-cellular scaffolding and transmembrane domains, our mAb (clone no. 136.20.1) detects CD19scFv within CAR expressing scFv derived from FMC63.

**Figure 4:**
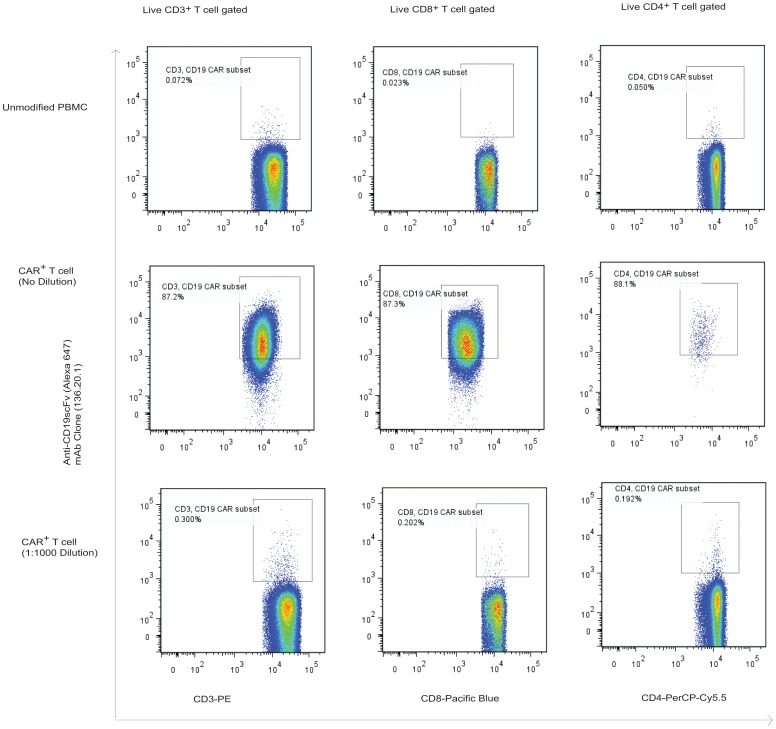
Sensitivity of clone no. 136.20.1 to detect CD19-specific CAR^+^ T cells in PBMC. CD19-specific T cells expressing CD19RCD28 were serially diluted in PBMC (1∶10 to 1∶10,000). (A) The upper panel shows background level signal in PBMC control. (B) Middle panel shows staining of CAR^+^ T cells in undiluted sample. (C) Bottom panel shows lowest detection level (1∶1,000) of CAR^+^ T cells in PBMC. Data acquisition by FACSCaliber and plots were analyzed using FlowJo software. Shown in picture are representative flow cytometry data obtained from two independent experiments.

The anti-CD19scFV mAb (clone no. 136.20.1) could be used in multi-parameter flow cytometry analysis to characterize CAR^+^ cells selectively propagated on K562-derived aAPC [14], [16]. CAR-modified T cells were numerically expanded in the presence of irradiated aAPC and cytokines (IL-2 and IL-21) for 4 weeks as previously reported [16]. The anti-CD19scFV mAb (clone 136.20.1) could detect both CD4^+^ and CD8^+^ CAR^+^ T cells (Fig. 3C). We assessed the limit of detection using a spiking experiment. As shown in Fig. 4, we were able to detect 1 CD19-specific CAR^+^ T cell in 1,000 PBMC using anti-CD19scFV tagged to a AlexaFluor 647 dye. The assay was validated in PBMC obtained from two individual donors by an independent laboratory (Immune Monitoring Core lab) at MDACC (Representative data along with dilution schematic and gating strategy are provided in Fig. S5. The anti-CD19scFV mAb could help in detecting the presence of CAR^+^ T cells in patients after infusion (Fig. S4 demonstrating the detection of CAR^+^ T cells with central memory immunophenotype in PBMC). Our FDA-approved trials (IND# 14193, 14577, 14739) is currently enrolling patients and we anticipate clone no. 136.20.1 being a valuable tool to help investigate the persistence and biodistribution CD19RCD28^+^ T cells recovered from blood and tissues after infusion.

**Figure 5:**
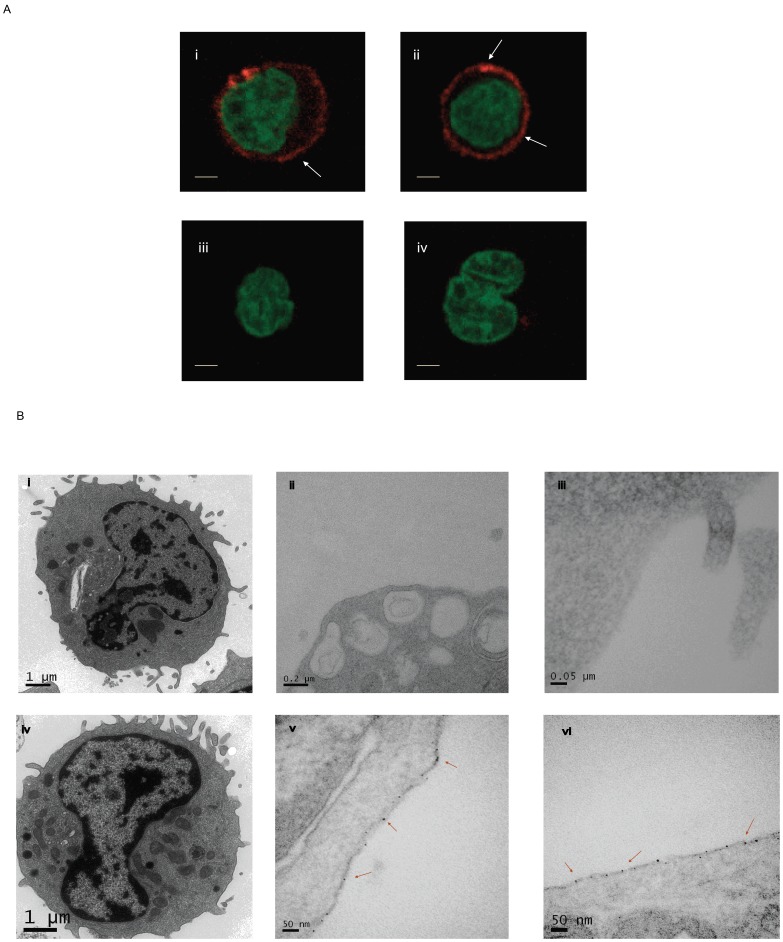
Localization of CD19RCD28 CAR on the surface of genetically modified T cells. (A) Genetically modified CAR^+^ T cells (expressing CD19RCD28) and unmodified control T cells were fixed using paraformaldehyde, stained with AlexaFluor 647-conjugated mAb clone no. 136.20.1 and then spread on glass slides using cytospin. Confocal images were acquisitioned by Leica microscope (60X magnification). Upper panels (i and ii) showed surface distribution of CAR molecules and bottom panels (iii and iv) showed no staining in unmodified control T cells. Nuclear staining was by DAPI (pseudo-color green). The bar (1 µm) on images indicates scale. (B) TEM images showing staining of gold-labeled nanoparticles conjugated to mAb clone no. 136.20.1. Upper row: Unmodified control T cells. (i) 120 nm thin section; 15 k magnification stained with uranyl acetate. (ii) 120 nm thin section; 75 k magnification, and (iii) 120 nm thin section; 200 k magnification. No gold particles were appreciated in these CAR^neg^ T cells. Bottom row: CAR^+^ T cells with enforced expression of CD19RCD28. (iv) 120 nm thin section stained with uranyl acetate; 20 k magnification. (v) 120 nm thin section; 200 k magnification, and (vi) 80 nm thin section 200K magnification. Arrow heads on plates v and vi indicate surface distribution of gold-labeled nanoparticles attached to CAR molecules. The bar on images indicates scale.

Persistence of adoptively transferred CAR^+^ T cells in patients correlates with efficacy in eradicating tumor. We desired that clone no. 136.20.1 be used in correlative studies pertaining to clinical trials that infuse CAR^+^ T cells and thus wanted to check if this mAb could detect CAR^+^ cells preserved in various harsh organic solvent fixatives. We demonstrated that CARs could be detected by immunocytochemistry and TEM. At day 28 of culture CD19-specific CAR^+^ T cells were harvested and fixed by paraformaldehyde. Fig. 5A shows anti-CD19scFv mAb when used at a concentration of 1∶500 can establish surface localization of CARs in genetically modified T cells by immunocytochemistry. We have also established the utility of this mAb in immunohistochemistry using CAR^+^ T cells fixed in neutral buffer formalin (Fig. S6) and we are currently working to detect CAR^+^ T cells in bone marrow of patients infused with CD19-specific CAR^+^ T cells. To detect CAR molecules on surface of genetically engineered T cells by electron microscopy, mAb (clone no. 136.20.1) was tagged to gold-labeled nanoparticles. Control unmodified T-cell sections are shown in plate (Fig. 5B i–iii). The size of the conjugated nanogold (1.4 nm) precluded detection within thick sections of CAR^+^ T cells (Fig. 5B plate iv). Thus, we performed a silver enhancement process and could view CARs as detected by clone no. 136.20.1 conjugated nanoparticles within 100 nm thin sections (Fig. 5B v and vi). The silver enhancement was performed on non-stained samples as uranyl acetate staining prevented distinguishing nanoparticles from other cellular granules. These studies establish the utility of clone no. 136.20.1 to detect CAR^+^ T cells in fixed sections.

### Anti-CD19scFv mAb can Block the Effector Function of CAR^+^ T Cells

**Figure 6:**
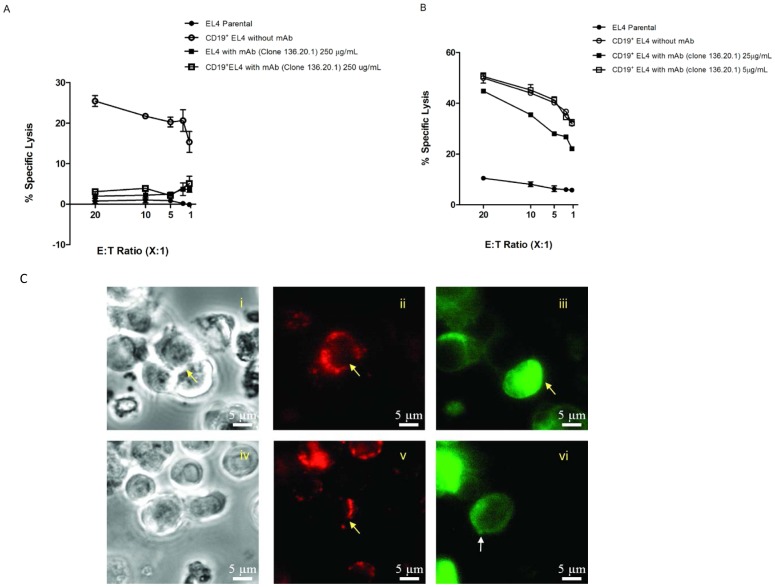
Clone no. 136.20.1 inhibits CAR^+^ T-cell effector function. (A) Data from CRA shows inhibition of tumor cell lysis in CD19^+^ EL4 cells when CD19-specific CAR^+^T cells were blocked with clone no. 136.20.1 (B) Dose response curve showing clone no. 136.20.1-mediated inhibition of lysis of CD19^+^ EL4 target cells. (C) Images obtained from VTLM showing CD19-specific CAR^+^ T cells co-cultured with CD19^+^ Daudiβ_2_m expressing EGFP and stained with either clone no. 136.20.1 conjugated to AlexaFluor-647 or PE-conjugated anti-Fc mAb that recognizes CAR scaffold. Images show two separate focal planes (at 2 and 90 minutes respectively). Upper panels (i-iii) reveal coculture of Daudiβ_2_m target cells with CAR^+^ T cells in the presence of Fc-specific antibody; Lower panels (iv-vi) reveal coculture of Daudiβ_2_m target cells with CAR^+^ T cells in the presence of clone no. 136.20.1. Panels (i-iv) show phase contrast images of CAR^+^ T cells along with Daudiβ_2_m. Panels (ii-v) show formation of immunological synapse (yellow arrow heads) when CD19-specfiic CAR^+^ T cells attack tumor cells. Panel (iii) shows killing of CD19^+^ tumor cell by CD19-specific CAR^+^ T cell as observed by formation of green fluorescence blub. Panel (vi) shows intact Daudiβ_2_m cell when the CAR^+^ T cells were blocked by clone no. 136.20.1 and no killing was observed even when effector T cells engaged the tumor cells for more than 6 hours. Shown in figure are data from 3 independent experiments. Movies are available online as supplementary files (Movie S1 and Movie S2).

We evaluated functional properties of anti-CD19scFv mAb (clone no. 136.20.1) based on its ability to prevent lysis mediated by CD19-specific CAR^+^T cell. Previously we have developed and validated an approach for ex vivo expansion of genetically modified CAR^+^T cells on artificial antigen presenting cell (K562 clone no. 4). CAR^+^ T cells receive proliferative signals from the TAA (CD19) coordinated with co-stimulatory molecules (CD86, CD137L, membrane bound IL15) cloned onto the surface of K562 cells [13], [14], [16]. The CD19-specific CAR^+^ T cells used in this study were propagated on same K562-derived aAPC as published previously. A standard 4 hour CRA was performed using mouse T-cell lines EL4 (parental) and CD19^+^EL4 as the tumor target. The percentage specific lysis was calculated using CD19-specific CAR^+^ T cells bound to CD19scFv mAb or CD19-specific CAR^+^ T cells alone. Fig. 6A shows that anti-CD19ScFv mAb completely inhibits CAR-dependent killing (at 250 µg/mL) by blocking the ability of CAR to dock to the TAA. However, at 25 µg/mL the anti-Id inhibited killing at 30% maximum and there was little or no effect when the mAb concentration was reduced to 5 µg/mL (Fig. 6B). We repeated the experiment in two other B-cell lines (Daudiβ_2_m and NALM-6) to validate that inhibition of lysis could be achieved in alternate B-cell tumors. The application of anti-CD19scFv at 250 µg/mL reduced the cytolytic activity to 18% in Daudiβ_2_m and 32% in NALM-6 (Fig. S7). We hypothesize that the mechanism of lysis may not be same in all these tumor cell lines and hence the degree of inhibition differed. To investigate the inhibitory effect of anti-CD19scFv mAb in real time, we used VTLM to serially monitor the killing process in a co-culture experiment involving Daudiβ_2_m as tumor target and CD19-specific CAR^+^ T cells as effectors. As control, we used a Fc-specific F(ab′)_2_ fragment of an antibody that bound to the scaffolding of CD19RCD28. CAR^+^ T cells continued lyse tumor cells when the effector cells were bound by this control antibody (Fig. 6 i–iii; Movie S1). In contrast, CAR^+^ T cells were unable to kill target cells when bound by anti-CD19scFv mAb (Fig. 6 iv–vi; Movie S2). We reasoned that blocking by clone no. 136.20.1 prevented triggering of T cells which supports our claim that this mAb binds within the scFv domain of the CAR.

## Discussion

We developed and validated a mAb that recognizes scFv region of a CD19-specific CAR derived from anti-human CD19 mAb FMC63. Previously, CARs expressed on the T-cell surface were detected by antibodies directed against epitope tags or spacer domains incorporated in the extracellular domain. For example, the CAR recognizing prostate specific membrane antigen (PSMA) and erbB2 were detected by V5-specific or myc-specific mAbs [21], [22]. Spacer or scaffolding domains appending the scFv within the CAR from the cell surface can be derived from the hinge/CH_2_–CH_3_ domain of IgG_1_ [23] or IgG_4_ (Fig. 1A). Commercially-available antibodies detecting Fc regions can detect these CARs [1], [24], [25] but there is the potential for cross-reactivity against immunoglobulin especially when genetically modified T cells are to be assayed from patient-derived PBMC. Alternative CAR designs that are non-immunogenic [26], avoid using a CH_2_–CH_3_ domain for concern of binding to IgG Fc gamma receptors [27], and employ scaffolding domains that are smaller than the Fc region, may improve the therapeutic potential of genetically modified T cells. Thus, reagents such as mAb clone no. 136.20.1 that can directly detect the scFv region of the CAR may have increasing utility. Indeed, the anti-idiotype mAb we developed for detection of CD19-specific CAR^+^ T cells provides a methodology to generate reagents to detect CARs targeting TAAs alternative to CD19.

The application of our anti-CD19scFv mAb to enhance correlative studies has been demonstrated in studies following the fate of infused CAR^+^ T cells (ClinicalTrials.gov number NCT01029366, University of Pennsylvania). The persistence and immunophenotype of infused CD19-specific CAR^+^T cells were established in patients with CLL using fluorescent-conjugated clone no. 136.20.1 [5].

Most CARs developed to redirect T-cell specificity to TAAs employ scFvs derived from mouse mAbs [28]. Anti-idiotypic mAbs that bind CARs have been previously reported, for example anti-Leu Y or anti-GD2 [29], [30]. The idiotype specific mAb reported here has utility beyond detecting the CAR on T cells as monitoring host immune response targeting the scFv is needed to inform on the potential for immune-mediated clearance of the infused CAR^+^ T cells.

Hence, it may be necessary to determine if a recipient’s immune response is directed against the scFv region and thus can interfere with binding of the CAR^+^ T cells to the TAA. In a recent study, adoptively transferred antigen specific CAR^+^ T cells were shown to induce humoral response as detected by circulating anti-Id antibodies (up to 1 µg/mL) in infused patients [26]. Significantly, an anti-idiotypic response was found to neutralize CAR binding carbonic anhydrase IX (CAIX) that compromised the clinical utility of this immunotherapy. In this context, we determined that mAb clone no. 136.20.1 could inhibit the effector function of CD19-specific CAR^+^ T cells. In the clinical setting, the anti-idiotype antibody response that interfered with CAR^+^ T cells occurred at mAb concentrations lower than we used to block lysis in CRA and VTLM. This could be due to different dynamics in an in vitro assay system where the CAR^+^ T cells act rapidly on tumor targets expressing high loads of antigen. An ability to block the effector functions triggered by CAR may have therapeutic implications as mAb clone no. 136.20.1 could be administered to patients who receive genetically modified T cells, to block effector functions of infused cells and ameliorate symptoms in patients that experience adverse events (such as cytokine storm) stemming from the synchronous activation of CAR^+^ T cells by resident CD19^+^ cells. [7].

In summary, we report an anti-idiotype mAb that binds to the scFv derived from anti-human CD19 mAb FMC63 which can detect genetically modified CD19-specific T cells and interferes with killing of CD19^+^ target cells. This reagent will be helpful to multiple investigators involved in developing and implementing gene therapy trials infusing CAR^+^ T cells.

## Supporting Information

Figure S1 **Schematic describes the steps for generating clone no. 136.20.1 mAb by L-cell immunization.** DNA plasmid encoding the scFv as immunogen was introduced into L cells by an electroporator device (Lonza). L cells stably expresses the transgenes under G418 drug selection. BALB/c mice were immunized in the foot pads and draining lymph nodes were harvested to collect lymphocytes. Candidate hybridoma clones were isolated and expanded for isolation of mAb that could detect CD19-specific CAR that employs scFv region derived from FMC63.
(TIF)

Figure S2 **Specificity of anti-CD19scFv mAb (clone no. 136.20.1) towards CD19-specific CAR^+^ T cells.** A panel of CAR^+^ T cells were collected and stained with a commercial Fc-specific antibody (goat Fab_2_ anti-human Fc gamma-PE) followed by staining with anti-CD19scFv mAb-Alexa 488. Shown in image are (A) Isotype control, (B) CD19RCD28 CAR^+^ T cells, (C) CD123-specific T cells (expressing CD123RCD28mZ-CAR), (D) CD33-specific T cells (expressing CD33RCD28z/Neo-CAR from Dr. Dean Lee), (E) ROR1-specific T cells (expressing ROR1RCD137mz-CAR), and (F) HERV-K-specific T cells (expressing HERV-K-CD28z CAR). All cells are co-stained with Fc-specific antibody as well as clone no. 136.20.1. Only T cells expressing the CD19RCD28 CAR co-stained with both Fc-specific antibody and clone no. 136.20.1.
(TIF)

Figure S3 **Flow cytometry analysis of T cells (produced at Baylor College of Medicine) transduced with a retroviral vector to express a CD19-specific CAR that activates via CD28 and CD3-zeta endodomains** [**31**] **.** T lymphocytes were stained with anti-Fc-γ cyanine-Cy5-conjugated mAb, which recognizes the IgG_1_-CH_2_CH_3_ component of this CAR or with anti-CD19scFv Alexa Fluor 647-conjugated mAb (clone no. 136.20.1). Shown in figure are percentage of CAR^+^ T cells detected by anti-Fcγ (67%), anti-CD19scFv mAb (66%) against matched isotype control.
(TIF)

Figure S4 **Flow cytometry analysis of T cells transduced with a lentiviral vector at City of Hope encoding CD19R-zeta that contains CD19scFv, IgG_4_ hinge/Fc stalk, CD4 trans-membrane, and CD3-zeta intracellular domains** [**32**] **.** (A) Histogram plot shows comparable level of CAR expression on surface of genetically modified T cells as detected by Alexa Fluor 647-conjugated clone no. 136.20.1 mAb (76%) and anti-Fc streptavidin-PE antibodies (61%) (B) Central memory T cells, defined as CD62L^+^CD45RO^+^ (Tcm) were obtained by depletion of CD45RA^+^, CD4^+^, and CD14^+^ cells from PBMC of healthy donor and then by positive selection on anti-CD62L mAb biotin and anti-biotin beads on CliniMACS device. Enriched CD8^+^Tcm were transduced with CD19R-zeta lentiviral vector and expanded ex vivo. Live cells were identified by staining with 7-AAD and then gated on CD3-FITC (with anti-CD14-PE and anti-CD16-PE as dump antibodies), anti-CD19scFv CAR AlexaFluor 647 antibody was used for detection CD19-specific CAR^+^ T cell. Staining of CD8^+^Tcm derived CD19-specific CAR^+^ T-cell product diluted in unmodified PBMC (T cell product : unmodified PBMC ∼ 1∶5), with unmodified PBMC as negative control are shown.
(TIF)

Figure S5 **Flow Cytometry detection sensitivity of CD19-specific CAR^+^ T cells mixed with PBMC from healthy donors.** Shown are plots obtained from entire dilution range (1∶10–1∶10,000) of CAR^+^ T cells mixed with PBMC. Cells after mixing, were gated on live Aqua stain (Life Tech) lymphocytes (primary gate), followed by positive gating on CD3^+^, CD4^+^, CD8^+^ T cells (Fig. i, ii and iii respectively) (secondary gate), and then identified as co-staining with Alexa-Fluor 647-conjugated clone no. 136.20.1 mAb (tertiary gate). For each, CAR^+^ T cells within specific lymphocyte population are shown along with back-gating within original lymphocyte population.
(TIF)

Figure S6 **Immunohistochemical staining of a panel of T cells (CAR modified and unmodified control) in formaldehyde fixed and paraffin embedded sections.** (A) H&E staining of CAR^+^ T cells. (B) Absence of specific staining was observed in sections of CAR^neg^ control T cells stained with clone no. 136.20.1 mAb along with HRP-conjugated detection antibody, and counter stained with hematoxylene. (C) CD19-specific CAR^+^ T cells expressing CD19RCD28 stained with clone no. 136.20.1 mAb and peroxidase labeled secondary antibodies. Deposition of DAB on T cells indicates localization of CAR protein on the cell surface (Arrow).
(TIF)

Figure S7 **Inhibition of specific lysis mediated by CD19-specific CAR^+^ T effector cells in a CRA.** CD19-specific CAR^+^ T cells were incubated with clone no. 136.20.1 mAb at 250 µg/mL and then washed to remove unbound antibody. The effector cells were then co-cultured with ^51^Cr-labeled CD19^+^ tumor targets (NALM-6 and Daudiβ2m). Percentage specific lysis were calculated at different Effector:Target (E:T) ratios, for effector cells bound by clone no. 136.20.1 and effector cells only.
(TIF)

Table S1 **List of antibodies used for flow cytometry analysis in the study.**
(DOCX)

Movie S1 **CAR^+^ T cells were blocked with anti-Fc PE-conjugated antibodies and co-cultured with Daudiβ_2_m (2∶1) in a humidified chamber (37°C, 5% CO_2_) of Nikon Biostation IM.** Movie was made from frames taken at 2 minutes intervals for 2 hours (time on left corner of frame; h:min:sec) (5 µm scale). We show here a single CAR^+^ T cell (as detected by anti-Fc PE F(ab′)_2_, pseudo-red) engaging CD19^+^ tumor cells (EGFP^+^ Daudiβ_2_m, green fluorescence). Killing event is marked by fluorescence blub.
(AVI)

Movie S2 **CAR^+^ T cells were blocked with Alexa Fluor 647-conjugated clone no. 136.20.1 mAb and co-cultured as described for Movie S1.** Movie was made with frames taken at 2 minutes intervals for 11 hours (time on left corner of frame; hr:min:sec) (5 µm scale). Synapse was formed by the effector CAR^+^ T cell with the Daudi β_2_m (green fluorescent) target cells, but no lysis of tumor cells occurred even after the tumor and effectors were engaged for more than 6 hours.
(AVI)

Method S1 **Indirect ELISA to detect antibody titer and screen positive hybridoma clones.**
(DOCX)

Method S2 **Antibody purification and conjugation.**
(DOCX)

Method S3 **Immunohistochemistry.**
(DOCX)

## References

[1] Till BG, Jensen MC, Wang J, Chen EY, Wood BL, et al (2008) Adoptive immunotherapy for indolent non-Hodgkin lymphoma and mantle cell lymphoma using genetically modified autologous CD20-specific T cells. Blood 112: 2261–2271. 18509084 10.1182/blood-2007-12-128843 PMC2532803

[2] Pule MA, Savoldo B, Myers GD, Rossig C, Russell HV, et al (2008) Virus-specific T cells engineered to coexpress tumor-specific receptors: persistence and antitumor activity in individuals with neuroblastoma. Nat Med 14: 1264–1270. 18978797 10.1038/nm.1882 PMC2749734

[3] Morgan RA, Dudley ME, Rosenberg SA (2010) Adoptive cell therapy: genetic modification to redirect effector cell specificity. Cancer J 16: 336–341. 20693844 10.1097/PPO.0b013e3181eb3879 PMC6348476

[4] Kochenderfer JN, Wilson WH, Janik JE, Dudley ME, Stetler-Stevenson M, et al (2010) Eradication of B-lineage cells and regression of lymphoma in a patient treated with autologous T cells genetically engineered to recognize CD19. Blood 116: 4099–4102. 20668228 10.1182/blood-2010-04-281931 PMC2993617

[5] Kalos M, Levine BL, Porter DL, Katz S, Grupp SA, et al (2011) T cells with chimeric antigen receptors have potent antitumor effects and can establish memory in patients with advanced leukemia. Sci Transl Med 3: 95ra73. 10.1126/scitranslmed.3002842 PMC3393096 21832238

[6] Brentjens RJ, Riviere I, Park JH, Davila ML, Wang X, et al (2011) Safety and persistence of adoptively transferred autologous CD19-targeted T cells in patients with relapsed or chemotherapy refractory B-cell leukemias. Blood 118: 4817–4828. 21849486 10.1182/blood-2011-04-348540 PMC3208293

[7] Kochenderfer JN, Dudley ME, Feldman SA, Wilson WH, Spaner DE, et al (2012) B-cell depletion and remissions of malignancy along with cytokine-associated toxicity in a clinical trial of anti-CD19 chimeric-antigen-receptor-transduced T cells. Blood 119: 2709–2720. 22160384 10.1182/blood-2011-10-384388 PMC3327450

[8] O’Connor CM, Sheppard S, Hartline CA, Huls H, Johnson M, et al.. (2012) Adoptive T-cell therapy improves treatment of canine non-Hodgkin lymphoma post chemotherapy. Scientific Reports 2. DOI: 10.1038/srep00249. 10.1038/srep00249 PMC3278154 22355761

[9] Nicholson IC, Lenton KA, Little DJ, Decorso T, Lee FT, et al (1997) Construction and characterisation of a functional CD19 specific single chain Fv fragment for immunotherapy of B lineage leukaemia and lymphoma. Mol Immunol 34: 1157–1165. 9566763 10.1016/s0161-5890(97)00144-2

[10] Cooper LJN, Jena B, Bollard CM (2012) Good T cells for bad B cells. Blood 119: 2700–2702. 22442331 10.1182/blood-2011-12-398719

[11] June CH, Blazar BR, Riley JL (2009) Engineering lymphocyte subsets: tools, trials and tribulations. Nat Rev Immunol 9: 704–716. 19859065 10.1038/nri2635 PMC3412112

[12] Morgan RA, Dudley ME, Wunderlich JR, Hughes MS, Yang JC, et al (2006) Cancer regression in patients after transfer of genetically engineered lymphocytes. Science 314: 126–129. 16946036 10.1126/science.1129003 PMC2267026

[13] Singh H, Manuri PR, Olivares S, Dara N, Dawson MJ, et al (2008) Redirecting specificity of T-cell populations for CD19 using the Sleeping Beauty system. Cancer Res 68: 2961–2971. 18413766 10.1158/0008-5472.CAN-07-5600 PMC2424272

[14] Manuri PV, Wilson MH, Maiti SN, Mi T, Singh H, et al (2009) piggyBac transposon/transposase system to generate CD19-specific T cells for the treatment of B-lineage malignancies. Hum Gene Ther 21: 427–437. 10.1089/hum.2009.114 PMC2938363 19905893

[15] Hackett PB, Largaespada DA, Cooper LJ (2010) A transposon and transposase system for human application. Mol Ther 18: 674–683. 20104209 10.1038/mt.2010.2 PMC2862530

[16] Singh H, Figliola MJ, Dawson MJ, Huls H, Olivares S, et al (2011) Reprogramming CD19-specific T cells with IL-21 signaling can improve adoptive immunotherapy of B-lineage malignancies. Cancer Res 71: 3516–3527. 21558388 10.1158/0008-5472.CAN-10-3843 PMC3096697

[17] Torikai H, Reik A, Liu P-Q, Zhou Y, Zhang L, et al (2012) A foundation for universal T-cell based immunotherapy: T cells engineered to express a CD19-specific chimeric-antigen-receptor and eliminate expression of endogenous TCR. Blood 119: 5697–5705. 22535661 10.1182/blood-2012-01-405365 PMC3382929

[18] Huls MH, Figliola MJ, Dawson MJ, Olivares S, Kebriaei P, et al.. (2012) Clinical Application of Sleeping Beauty and Artificial Antigen Presenting Cells to Genetically Modify T Cells from Peripheral and Umbilical Cord Blood. J Vis Exp doi:10.3791/50070. 10.3791/50070 PMC3596954 23407473

[19] Whitlow M, Bell BA, Feng S-L, Filpula D, Hardman KD, et al (1993) An improved linker for single-chain Fv with reduced aggregation and enhanced proteolytic stability. Protein Engineering 6: 989–995. 8309948 10.1093/protein/6.8.989

[20] Porter DL, Levine BL, Kalos M, Bagg A, June CH (2011) Chimeric Antigen Receptor-Modified T Cells in Chronic Lymphoid Leukemia. New England Journal of Medicine 365: 725–733. 21830940 10.1056/NEJMoa1103849 PMC3387277

[21] Ma Q, Safar M, Holmes E, Wang Y, Boynton AL, et al (2004) Anti-prostate specific membrane antigen designer T cells for prostate cancer therapy. Prostate 61: 12–25. 15287090 10.1002/pros.20073

[22] Moeller M, Haynes NM, Kershaw MH, Jackson JT, Teng MW, et al (2005) Adoptive transfer of gene-engineered CD4+ helper T cells induces potent primary and secondary tumor rejection. Blood 106: 2995–3003. 16030195 10.1182/blood-2004-12-4906

[23] Savoldo B, Ramos CA, Liu E, Mims MP, Keating MJ, et al (2011) CD28 costimulation improves expansion and persistence of chimeric antigen receptor-modified T cells in lymphoma patients. J Clin Invest 121: 1822–1826. 21540550 10.1172/JCI46110 PMC3083795

[24] Kowolik CM, Topp MS, Gonzalez S, Pfeiffer T, Olivares S, et al (2006) CD28 costimulation provided through a CD19-specific chimeric antigen receptor enhances in vivo persistence and antitumor efficacy of adoptively transferred T cells. Cancer Res 66: 10995–11004. 17108138 10.1158/0008-5472.CAN-06-0160

[25] Park JR, Digiusto DL, Slovak M, Wright C, Naranjo A, et al (2007) Adoptive transfer of chimeric antigen receptor re-directed cytolytic T lymphocyte clones in patients with neuroblastoma. Mol Ther 15: 825–833. 17299405 10.1038/sj.mt.6300104

[26] Lamers CH, Willemsen R, van Elzakker P, van Steenbergen-Langeveld S, Broertjes M, et al (2011) Immune responses to transgene and retroviral vector in patients treated with ex vivo-engineered T cells. Blood 117: 72–82. 20889925 10.1182/blood-2010-07-294520

[27] Hombach A, Hombach AA, Abken H (2010) Adoptive immunotherapy with genetically engineered T cells: modification of the IgG1 Fc ‘spacer’ domain in the extracellular moiety of chimeric antigen receptors avoids ‘off-target’ activation and unintended initiation of an innate immune response. Gene Ther 17: 1206–1213. 20555360 10.1038/gt.2010.91

[28] Jena B, Dotti G, Cooper LJ (2010) Redirecting T-cell specificity by introducing a tumor-specific chimeric antigen receptor. Blood 116: 1035–1044. 20439624 10.1182/blood-2010-01-043737 PMC2938125

[29] Rossig C, Bollard CM, Nuchtern JG, Merchant DA, Brenner MK (2001) Targeting of G(D2)-positive tumor cells by human T lymphocytes engineered to express chimeric T-cell receptor genes. Int J Cancer 94: 228–236. 11668503 10.1002/ijc.1457

[30] Rossig C, Bollard CM, Nuchtern JG, Rooney CM, Brenner MK (2002) Epstein-Barr virus-specific human T lymphocytes expressing antitumor chimeric T-cell receptors: potential for improved immunotherapy. Blood 99: 2009–2016. 11877273 10.1182/blood.v99.6.2009

[31] Vera J, Savoldo B, Vigouroux S, Biagi E, Pule M, et al (2006) T lymphocytes redirected against the kappa light chain of human immunoglobulin efficiently kill mature B lymphocyte-derived malignant cells. Blood 108: 3890–3897. 16926291 10.1182/blood-2006-04-017061 PMC1895462

[32] Cooper LJ, Topp MS, Serrano LM, Gonzalez S, Chang WC, et al (2003) T-cell clones can be rendered specific for CD19: toward the selective augmentation of the graft-versus-B-lineage leukemia effect. Blood 101: 1637–1644. 12393484 10.1182/blood-2002-07-1989

