# Supplementary figures and images for: Chimeric Antigen Receptor (CAR)-Specific Monoclonal Antibody to Detect CD19-Specific T Cells in Clinical Trials
Source: PLoS One. 2013 Mar 1;8(3):e57838. doi: 10.1371/journal.pone.0057838 (PMC3585808; doi:10.1371/journal.pone.0057838)

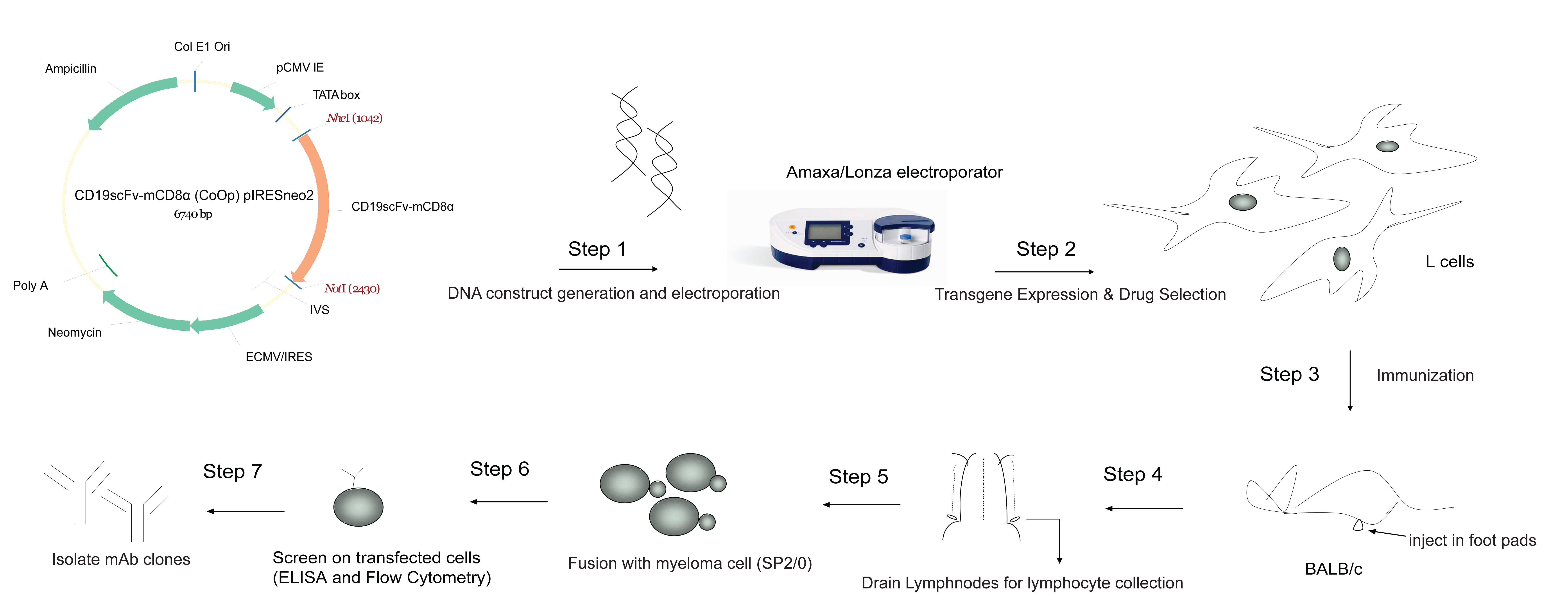

Supplement: Figure S1 — Schematic describes the steps for generating clone no. 136.20.1 mAb by L-cell immunization. DNA plasmid encoding the scFv as immunogen was introduced into L cells by an electroporator device (Lonza). L cells stably expresses the transgenes under G418 drug selection. BALB/c mice were immunized in the foot pads and draining lymph nodes were harvested to collect lymphocytes. Candidate hybridoma clones were isolated and expanded for isolation of mAb that could detect CD19-specific CAR that employs scFv region derived from FMC63. (TIF) [file pone.0057838.s001.tif]

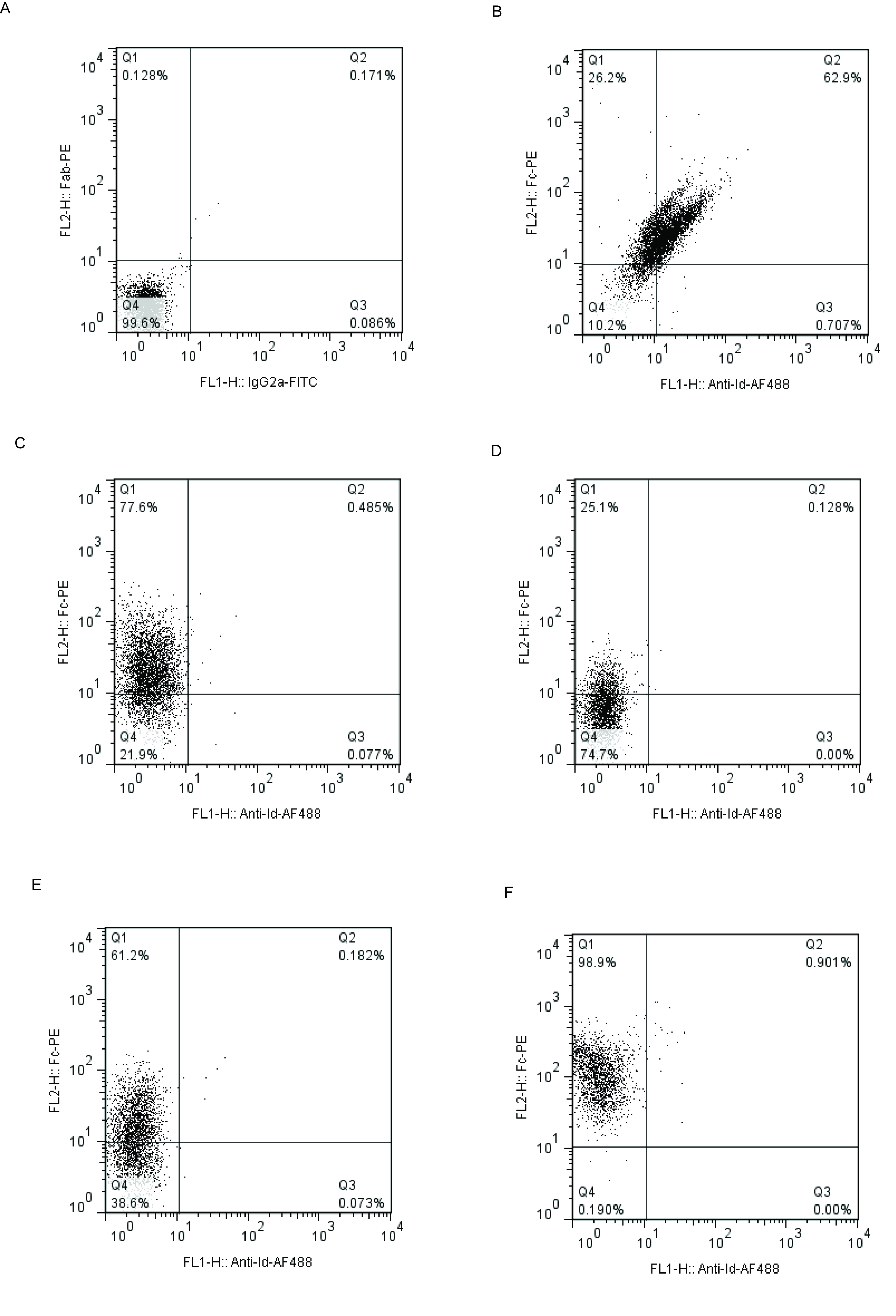

Supplement: Figure S2 — Specificity of anti-CD19scFv mAb (clone no. 136.20.1) towards CD19-specific CAR+ T cells. A panel of CAR+ T cells were collected and stained with a commercial Fc-specific antibody (goat Fab2 anti-human Fc gamma-PE) followed by staining with anti-CD19scFv mAb-Alexa 488. Shown in image are (A) Isotype control, (B) CD19RCD28 CAR+ T cells, (C) CD123-specific T cells (expressing CD123RCD28mZ-CAR), (D) CD33-specific T cells (expressing CD33RCD28z/Neo-CAR from Dr. Dean Lee), (E) ROR1-specific T cells (expressing ROR1RCD137mz-CAR), and (F) HERV-K-specific T cells (expressing HERV-K-CD28z CAR). All cells are co-stained with Fc-specific antibody as well as clone no. 136.20.1. Only T cells expressing the CD19RCD28 CAR co-stained with both Fc-specific antibody and clone no. 136.20.1. (TIF) [file pone.0057838.s002.tif]

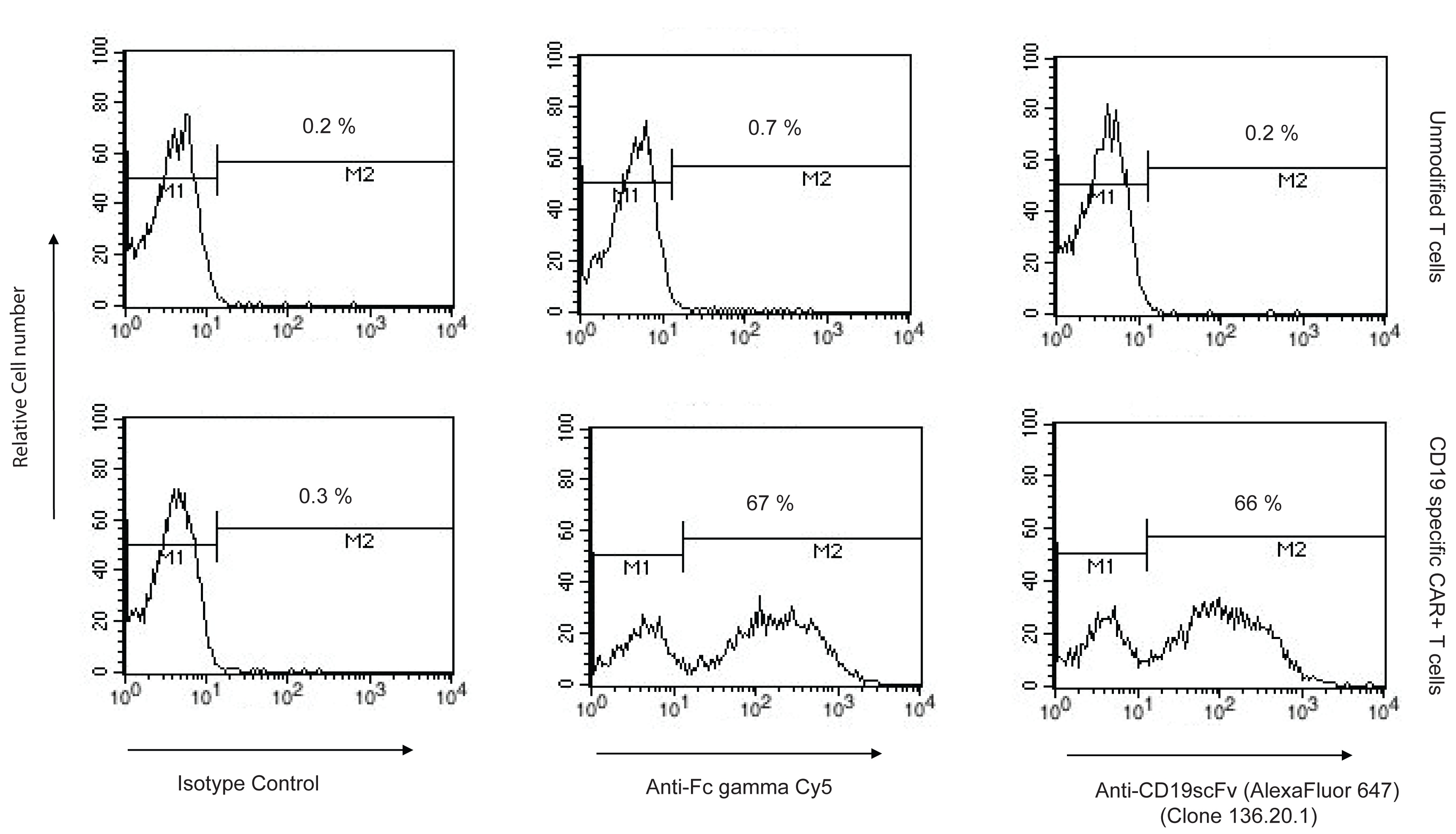

Supplement: Figure S3 — Flow cytometry analysis of T cells (produced at Baylor College of Medicine) transduced with a retroviral vector to express a CD19-specific CAR that activates via CD28 and CD3-zeta endodomains [31] . T lymphocytes were stained with anti-Fc-γ cyanine-Cy5-conjugated mAb, which recognizes the IgG1-CH2CH3 component of this CAR or with anti-CD19scFv Alexa Fluor 647-conjugated mAb (clone no. 136.20.1). Shown in figure are percentage of CAR+ T cells detected by anti-Fcγ (67%), anti-CD19scFv mAb (66%) against matched isotype control. (TIF) [file pone.0057838.s003.tif]

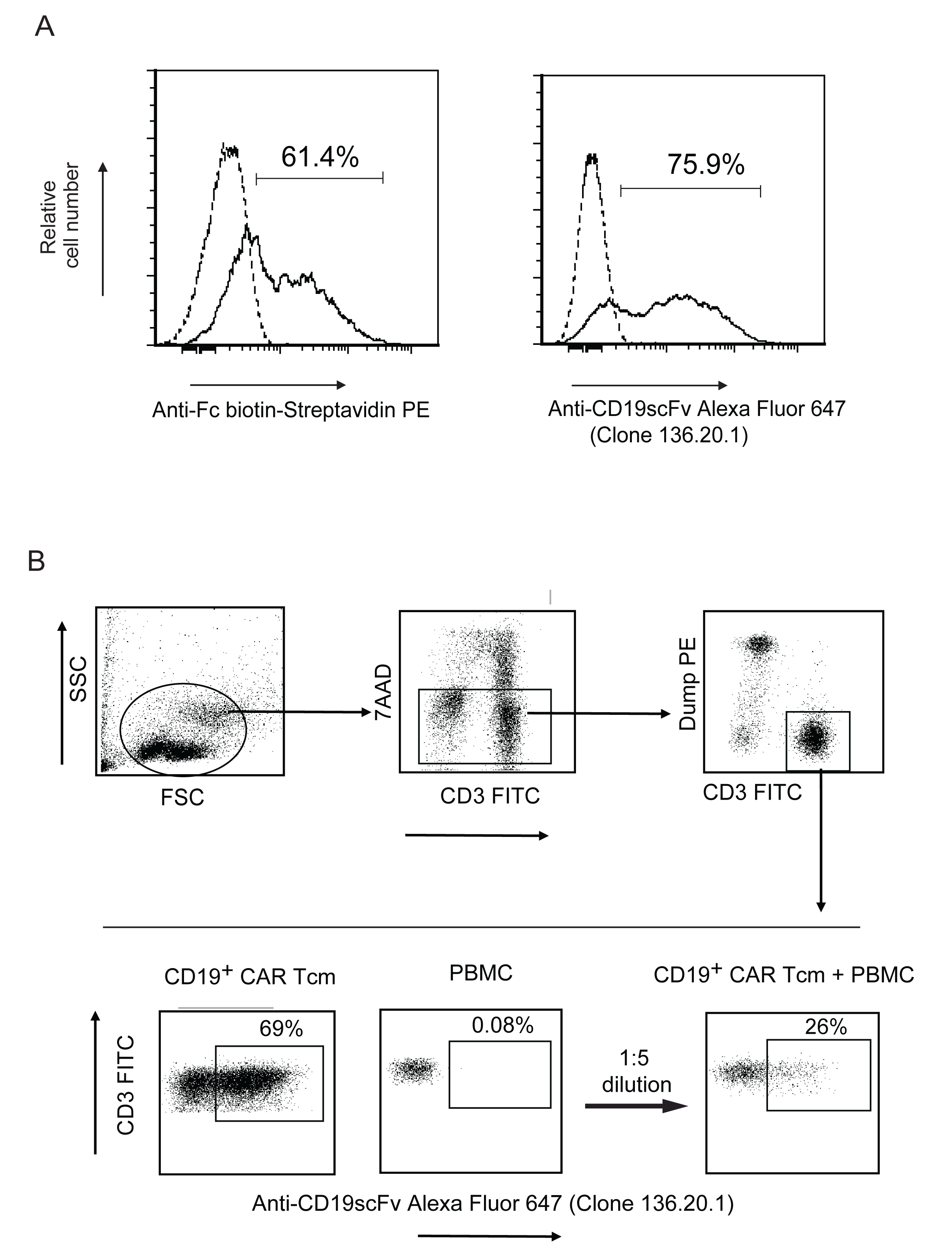

Supplement: Figure S4 — Flow cytometry analysis of T cells transduced with a lentiviral vector at City of Hope encoding CD19R-zeta that contains CD19scFv, IgG4 hinge/Fc stalk, CD4 trans-membrane, and CD3-zeta intracellular domains [32] . (A) Histogram plot shows comparable level of CAR expression on surface of genetically modified T cells as detected by Alexa Fluor 647-conjugated clone no. 136.20.1 mAb (76%) and anti-Fc streptavidin-PE antibodies (61%) (B) Central memory T cells, defined as CD62L+CD45RO+ (Tcm) were obtained by depletion of CD45RA+, CD4+, and CD14+ cells from PBMC of healthy donor and then by positive selection on anti-CD62L mAb biotin and anti-biotin beads on CliniMACS device. Enriched CD8+Tcm were transduced with CD19R-zeta lentiviral vector and expanded ex vivo. Live cells were identified by staining with 7-AAD and then gated on CD3-FITC (with anti-CD14-PE and anti-CD16-PE as dump antibodies), anti-CD19scFv CAR AlexaFluor 647 antibody was used for detection CD19-specific CAR+ T cell. Staining of CD8+Tcm derived CD19-specific CAR+ T-cell product diluted in unmodified PBMC (T cell product : unmodified PBMC ∼ 1∶5), with unmodified PBMC as negative control are shown. (TIF) [file pone.0057838.s004.tif]

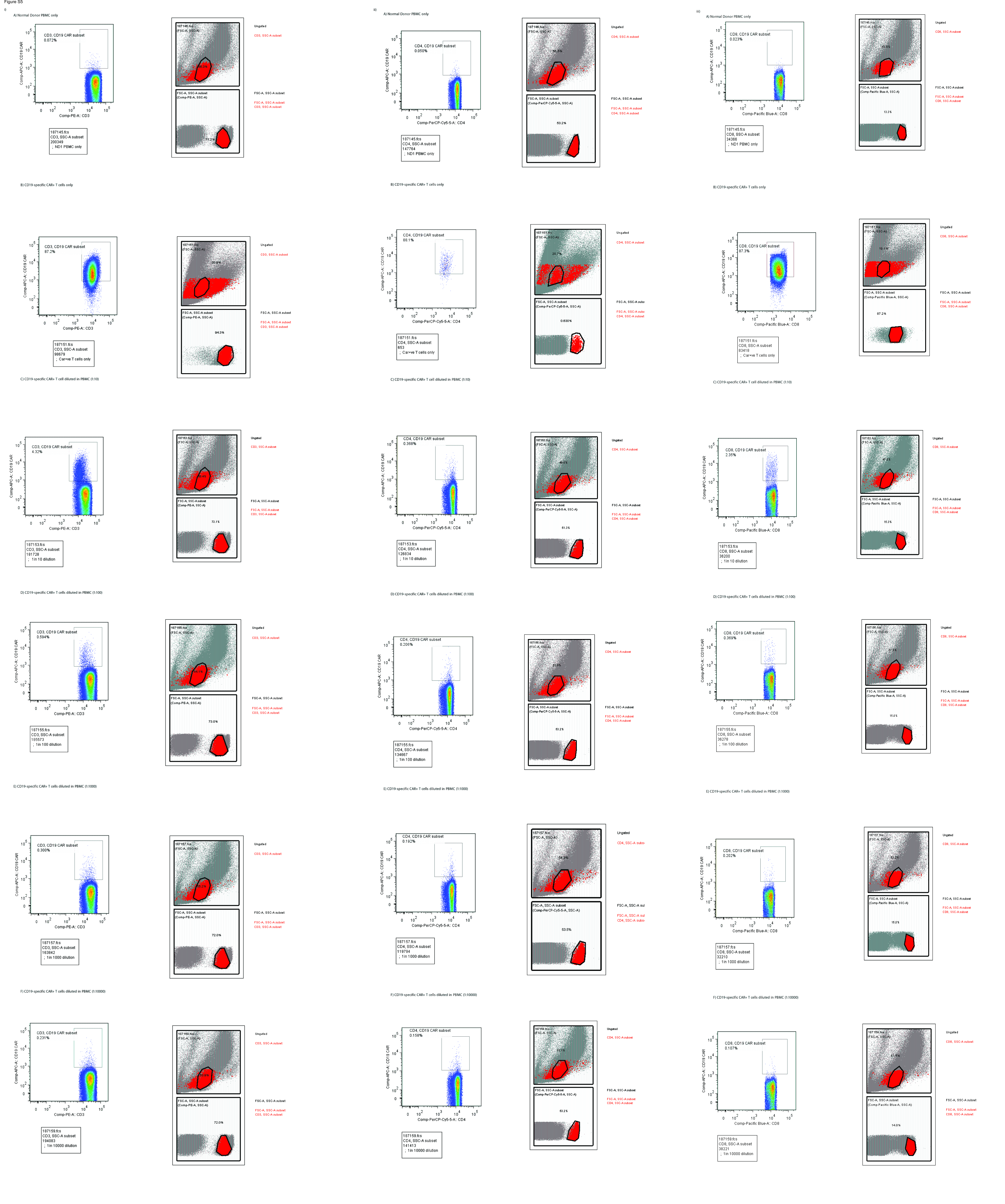

Supplement: Figure S5 — Flow Cytometry detection sensitivity of CD19-specific CAR+ T cells mixed with PBMC from healthy donors. Shown are plots obtained from entire dilution range (1∶10–1∶10,000) of CAR+ T cells mixed with PBMC. Cells after mixing, were gated on live Aqua stain (Life Tech) lymphocytes (primary gate), followed by positive gating on CD3+, CD4+, CD8+ T cells (Fig. i, ii and iii respectively) (secondary gate), and then identified as co-staining with Alexa-Fluor 647-conjugated clone no. 136.20.1 mAb (tertiary gate). For each, CAR+ T cells within specific lymphocyte population are shown along with back-gating within original lymphocyte population. (TIF) [file pone.0057838.s005.tif]

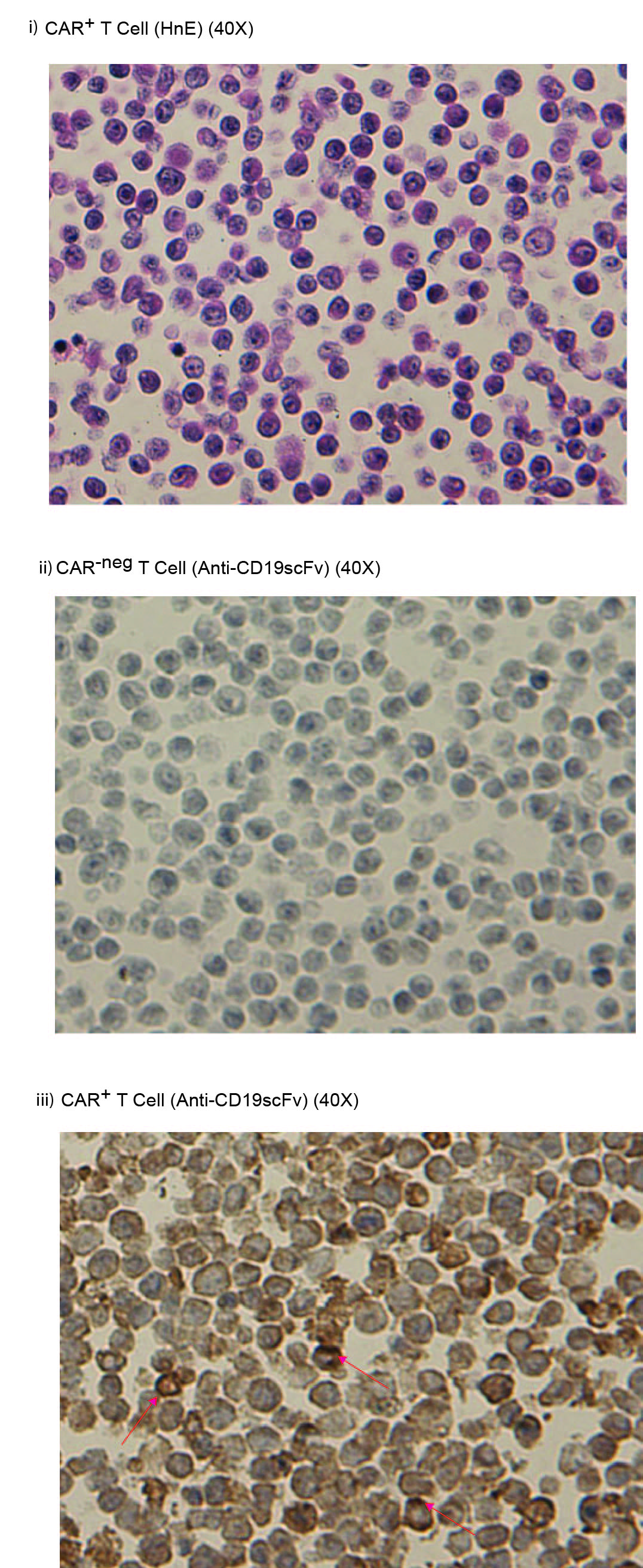

Supplement: Figure S6 — Immunohistochemical staining of a panel of T cells (CAR modified and unmodified control) in formaldehyde fixed and paraffin embedded sections. (A) H&E staining of CAR+ T cells. (B) Absence of specific staining was observed in sections of CARneg control T cells stained with clone no. 136.20.1 mAb along with HRP-conjugated detection antibody, and counter stained with hematoxylene. (C) CD19-specific CAR+ T cells expressing CD19RCD28 stained with clone no. 136.20.1 mAb and peroxidase labeled secondary antibodies. Deposition of DAB on T cells indicates localization of CAR protein on the cell surface (Arrow). (TIF) [file pone.0057838.s006.tif]

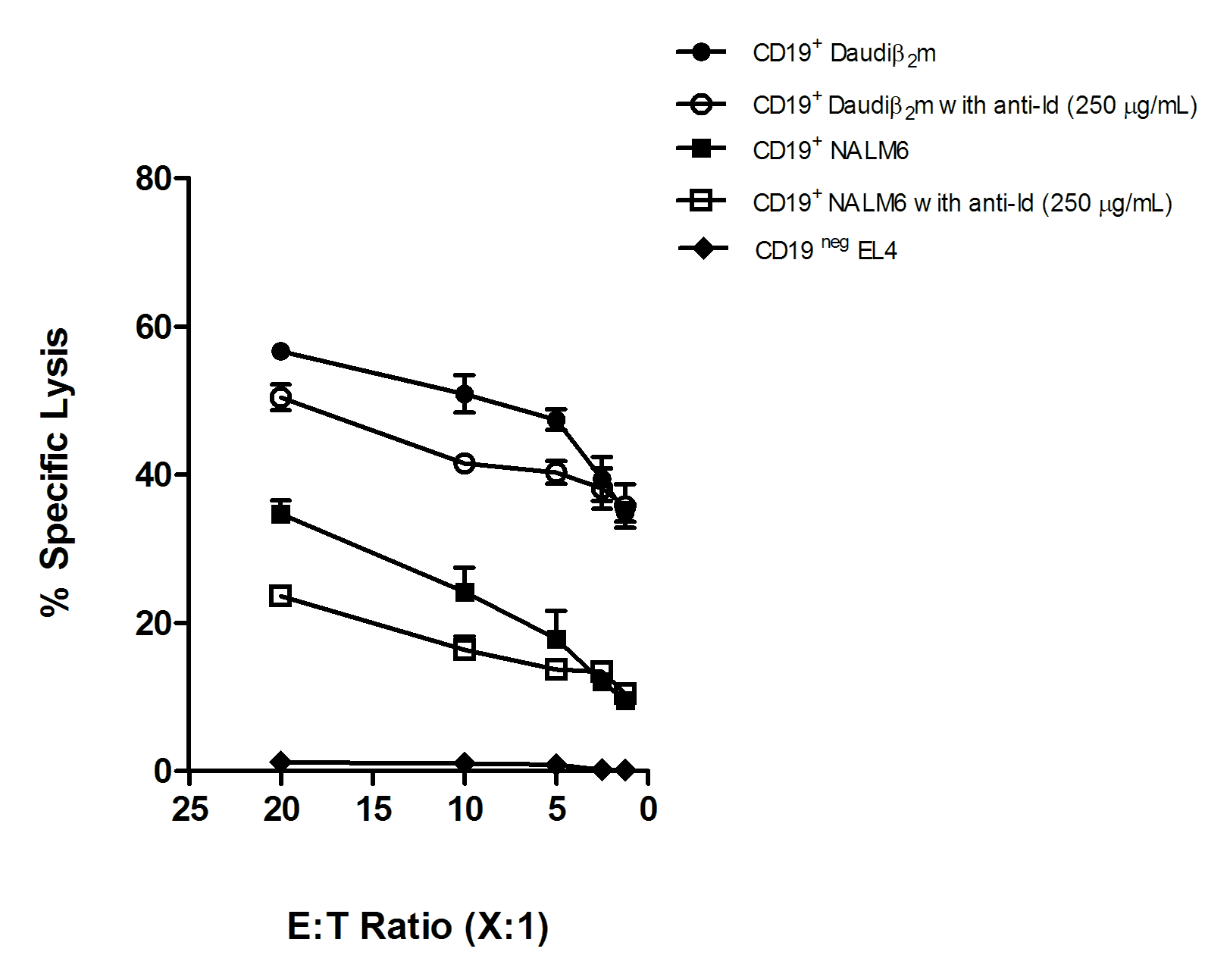

Supplement: Figure S7 — Inhibition of specific lysis mediated by CD19-specific CAR+ T effector cells in a CRA. CD19-specific CAR+ T cells were incubated with clone no. 136.20.1 mAb at 250 µg/mL and then washed to remove unbound antibody. The effector cells were then co-cultured with 51Cr-labeled CD19+ tumor targets (NALM-6 and Daudiβ2m). Percentage specific lysis were calculated at different Effector:Target (E:T) ratios, for effector cells bound by clone no. 136.20.1 and effector cells only. (TIF) [file pone.0057838.s007.tif]
