# Supplementary material for: Chimeric Antigen Receptor (CAR)-Specific Monoclonal Antibody to Detect CD19-Specific T Cells in Clinical Trials
Source: PLoS One. 2013 Mar 1;8(3):e57838. doi: 10.1371/journal.pone.0057838 (PMC3585808; doi:10.1371/journal.pone.0057838)
Supplement: Table S1 — List of antibodies used for flow cytometry analysis in the study. (DOCX) [file pone.0057838.s008.docx]

**Supplementary Table**

**Table S1:** Antibodies used for flow cytometry analysis in the study

| **Antibody** | **Amount (µL)** | | **Cat no.** | **Vendor** |
| --- | --- | --- | --- | --- |
| Rat Anti-mouse CD8α | | 1 | 553033 | Becton and Dickinson, San Jose, CA |
| Goat anti-mouse IgG Fcg fragment specific PE | 1 | | 115-096-071 | Jackson ImmunoResearch,  West Grove, PA |
| Goat F(Ab)2 anti-human IgGg R-PE | 2.5 | | H10104 | Invitrogen ,Carlsbad, CA |
| Anti-CD19^+^ CAR Fc specific (2D3) | 1 | | NA | Cooper Lab, MDACC |
| Anti-CD19^+^ CAR Idiotype 136.20.1- AlexaFluor-647AlexaFluor-488 | 1 | | NA | Cooper Lab, MDACC |
| Anti-CD3 PE | 1 | | 555333 | Becton and Dickinson, San Jose, CA |
| Anti-CD4 APC | 2.5 | | 555349 | Becton and Dickinson, San Jose, CA |
| Anti-CD4 PercP Cy5.5 | 2 | | 341654 | Becton and Dickinson, San Jose, CA |
| Anti-CD8 PercP Cy5.5 | 2 | | 341051 | Becton and Dickinson, San Jose, CA |
| Anti-CD8 Pacific blue | 1 | | 558207 | Becton and Dickinson, San Jose, CA |
