# Supplementary material for: Chimeric Antigen Receptor (CAR)-Specific Monoclonal Antibody to Detect CD19-Specific T Cells in Clinical Trials
Source: PLoS One. 2013 Mar 1;8(3):e57838. doi: 10.1371/journal.pone.0057838 (PMC3585808; doi:10.1371/journal.pone.0057838)
Supplement: Method S1 — Indirect ELISA to detect antibody titer and screen positive hybridoma clones. (DOCX) [file pone.0057838.s011.docx]

**Method S1: Indirect ELISA to detect antibody titer and screen positive hybridoma clones**

The assay was performed by coating flat-bottom well of a 96-well Medisorp plate (Thermo Scientific Nunc) with parental L cells or L cells transfected with CD19scFvmCD8α. Approximately 250,000 cells were coated per well in 50 µL of sodium phosphate buffer (pH 7.4). Plates were left overnight in laminar airflow hood for cell binding and drying and stored at -20^0^C until use. To block non-specific binding, 2% BSA in PBST (1X PBS containing 0.05% Tween-20) was dispensed at 300µL per well. Unbound cells were washed with PBST. Various dilutions of sera (1:100 to 1:3,000) or antibody supernatant (1:2-1:10) were allowed to bind with the coated cells/antigen for 1 hour at room temperature (RT). After washing with PBST, secondary antibody anti-mouse IgG Fc HRP (Sigma-Aldrich) was added at 1:5,000 dilution and incubated for 45 min at RT. Detection of the bound antibody was performed after addition of 1X TMB/H_2_O_2_ substrate (Sigma-Aldrich). Absorbance was measured at 450nm using a microplate reader (Victor 2030, Perkin Elmer). Cross reaction of immune sera and hybridoma supernatant with parental L cells was measured at OD_450_ and used as background controls. For transfected cells, wells showing OD_450_ values 3 times above background controls were marked positive. Random wells were viewed by microscopy before and after washing to ensure no loss of cells occurred during the process. The isotype of mAbs were determined using a mouse immunoglobulin isotyping kit (BD Biosciences). Positive clones were identified and expanded further in 1XHT (Sigma-Aldrich) supplement media. Sub-cloning was performed by standard limiting dilution method to obtain a monoclonal population. Monoclonal antibodies were purified using protein G or Protein A columns (GE Life Science).
