# Supplementary material for: Chimeric Antigen Receptor (CAR)-Specific Monoclonal Antibody to Detect CD19-Specific T Cells in Clinical Trials
Source: PLoS One. 2013 Mar 1;8(3):e57838. doi: 10.1371/journal.pone.0057838 (PMC3585808; doi:10.1371/journal.pone.0057838)
Supplement: Method S2 — Antibody purification and conjugation. (DOCX) [file pone.0057838.s012.docx]

**Method S2: Antibody purification and conjugation**

One CD19scFv specific clone (no. 136.20.1) was grown and expanded further *in vitro* or injected in mice for ascites production (Harlan Inc.). Monoclonal antibodies from the culture supernatant or ascites were obtained by standard protein G or A (GE Life Science) purification using 0.1M glycine (pH 2.7) as elution buffer. Affinity eluted and neutralized antibodies were dialyzed extensively against PBS and subjected to flow cytometry analysis for final validation and use in cell culture assays. Purified antibodies were conjugated to different fluorochromes *viz*., Alexa Fluor 488 and Alexa Fluor 647 (Molecular Probes-Invitrogen) using commercial available kit that exploits the principle of conjugation through succinimidyl ester moiety to primary amines of proteins/ immunoglobulins to form stable dye–protein conjugates. Fluorochrome conjugated antibodies were subjected to gel filtration chromatography to remove unbound dye. For transmission electron microscope (TEM) studies antibodies were conjugated to gold nanoparticles. In brief, antibodies were reduced by adding 1mL of purified mAb (2 mg/mL) to 6mg of mercaptoethylamine hydrochloride (MEA) (Thermoscientific-Pierce) and then purified on a gel filtration column MatrexGH25 (Millipore, Billerica, MA). Reduced antibodies were labeled with monomaleimido Nanogold^TM^ (Nanoprobes) as per manufacturer’ instructions and purified on a gel filtration column Superdex-75 (Sigma-Aldrich) to remove unbound Nanogold^TM^. The final concentration of the purified antibodies was measured by a BCA kit (Thermoscientific Pierce).
