# Supplementary material for: Chimeric Antigen Receptor (CAR)-Specific Monoclonal Antibody to Detect CD19-Specific T Cells in Clinical Trials
Source: PLoS One. 2013 Mar 1;8(3):e57838. doi: 10.1371/journal.pone.0057838 (PMC3585808; doi:10.1371/journal.pone.0057838)
Supplement: Method S3 — Immunohistochemistry. (DOCX) [file pone.0057838.s013.docx]

**Method S3: Immunohistochemistry**

CD19-specific CAR^+^T cells were propagated as described in Materials and methods. Cells were harvested, washed in 1X PBS and then fixed in formalin. 5 µM paraffin embedded sections were made and stained. In brief, sections were blocked initially with 3% hydrogen peroxide and whole goat sera blocking reagent for 5 minutes, followed by incubation with primary antibody anti-Id CAR idiotype for 30 minutes. Biotinylated goat anti-mouse IgG-HRP was used as secondary antibody at 1:500 for 30 minutes, followed by incubation with streptavidin-HRP for 15 minutes. Cells were localized with DAB (diaminobenzidine) for 5 minutes. Slides were counterstained with Mayer's hematoxylin for 5 minutes, dehydrated and mounted with cover slip. Photographs were taken with the help of a Leica microscope.
